# New Photoresponsive Poly(meth)acrylates Bearing Azobenzene Moieties Obtained via ATRP Polymerization Exhibiting Liquid-Crystalline Behavior

**DOI:** 10.3390/polym13132172

**Published:** 2021-06-30

**Authors:** Piotr Cieciórski, Paweł W. Majewski, Elżbieta Megiel

**Affiliations:** Faculty of Chemistry, University of Warsaw, Pasteura 1 Str., 02-093 Warsaw, Poland; p.cieciorski@uw.edu.pl (P.C.); pmajewski@chem.uw.edu.pl (P.W.M.)

**Keywords:** photoresponsivity, molecular switch, azobenzene, liquid crystals, controlled radical polymerization, ATRP

## Abstract

Here, we report our studies on photoresponsive poly(meth)acrylates containing azobenzene groups connected to a polymer backbone via a short methylene linker. A series of side-chain azobenzene polymers was synthesized via the atom transfer radical polymerization (ATRP) technique using several catalytic systems. The polymers synthesized under the optimized conditions were characterized by a narrow polydispersity (*Đ* ≤ 1.35), and they underwent a reversible transformation of their structure under light illumination. The fabricated polymers can store and release energy accumulated during the UV-light illumination by the thermal cis-trans isomerization of the chromophore groups. The enthalpy of the process (determined from DSC) was relatively high and equaled 61.9 J∙g^−1^ (17 Wh∙kg^−1^), indicating a high potential of these materials in energy storage applications. The liquid-crystalline behavior of the synthesized poly(meth)acrylates was demonstrated by the birefringent optical textures as observed in thin-films and X-ray scattering studies.

## 1. Introduction

Stimuli-responsive polymers belong to the group of smart materials that can adapt to surrounding conditions via a proper response to the stimulus [1]. Such stimuli-responsive polymeric materials change their properties drastically on receiving an external stimulus, for instance, the alteration of temperature, pH, treatment of solvent, or irradiation. Polymers that change properties due to irradiation, also called photoresponsive polymers (PRPs), are especially intensively studied due to the wide range of their possible applications in electronics, optics, designing of sensors and solar-thermal fuels, medicine, robotics, and many others [2,3]. The PRPs containing photochromic units undergo a reversible isomerization upon irradiation. As a result of the isomerization, the material properties, including polarity and absorption spectra, can be tuned reversibly.

PRPs with azobenzenes (AZs) as photochromic units are one of the most intensively studied. AZs undergo a reversible trans- to cis-isomerization of a double bond between nitrogen atoms (–N=N–) upon light irradiation. The trans isomer is nonpolar, and upon UV radiation it is transformed to the polar cis isomer. The reverse process can be induced thermally or by irradiation with visible light [4]. Thus, the AZs and PRPs containing such moieties can be used as photoswitches in advanced functional nanosystems such as light-driven motors, machines, and actuators [5,6]. It is noteworthy that azopolymers, when compared with AZs, are mechanically resilient and have hierarchical structures, allowing for the construction of novel materials with advanced architecture. Polymers containing azobenzene moieties exhibit many interesting photoresponsive properties, such as photoinduced orientation and photocontrolled self-assembly behavior. Notably, the incorporation of AZs molecules into a polymer matrix gives them also better processability [7].

There are two main types of homopolymers, with AZs groups being the first type, depending on their incorporation in a polymer chain. They can be grafted on a polymer chain or incorporated into repeating units (side-chain polymers) [8]. The second type usually provides a better photoresponsiveness of the obtained azobenzene-polymers due to the higher density of photochromic groups in a polymer chain [7]. Such multi-azobenzene polymers exhibit pronounced changes in their molecular structure under light radiation, leading to a macroscopic response, i.e., bending, torsional motion, or actuation of the material [9]. Significant changes in their glass transition temperatures (*T_g_*) under light irradiation can be observed for such polymers. Recently, Wu and coworkers showed that light could modulate the *T_g_* of AZ-containing polyacrylates, and they reported a light-induced, reversible solid-to-liquid transition [10]. They proved that the solid polymer with a *T_g_* above room temperature becomes liquid only due to light illumination (365 nm). It is noteworthy that the photoswitching of *T_g_* is a promising approach that can be used to create self-healing polymeric materials [10].

Properties of PRPs strictly depend on their molecular weight and polydispersity. Therefore, choosing a proper synthesis method to control these parameters is crucial to obtain smart material with the desired responsivity. Atom transfer radical polymerization (ATRP) is an excellent tool for constructing advanced functional polymers and systems with precisely defined architecture and molecular weight [11,12,13,14]. However, the synthesis of narrowly dispersed PRPs with azobenzenes via the ATRP technique is challenging. First of all, AZ moiety in a monomer molecule gives a steric hindrance, causing a low polymerization rate (inhibition effect). Furthermore, the catalytic complexes typically used in the ATRP could be blocked via irreversible binding with monomers containing AZ groups. [8] Therefore, an optimization of polymerization conditions, initiator, and the catalytic system’s composition is necessary to achieve the narrow dispersity of synthesized polymers for high monomer conversions.

There are only a few reports on the successful ATRP synthesis of side-chain azobenzene poly(meth)acrylates. Lu and coworkers reported the successful polymerization of several (meth)acrylates containing AZ groups via the ATRP technique. Namely, they used 4-(4-nitrophenyldiazenyl)phenyl acrylate (P-NPAPA) and 4-(4-methoxyphenyldiazenyl)phenyl acrylate (P-MPAPA) in the synthesis of homopolymers using *N*,*N*,*N*′,*N*″,*N*″-pentamethyldiethylenetriamine (PMDETA)/CuBr as a catalytic system and ethyl 2-bromoisobutyrate (EBriB) as an initiator in dry cyclohexanone [15]. The azobenzene moiety in the monomers was substituted by methoxyl or nitro moiety in para position and directly connected with the carboxyl group of polymer backbone (without any linker) or separated from the polymer backbone by an alkoxyl group built of two or six carbon atoms. The synthesized polymers presented relatively narrow dispersity (polydispersity index, *Đ* < 1.3). However, it was impossible to achieve high monomer conversion under such conditions even for a very long time of polymerization (40% conversion after 196 h). Additionally, the molecular weights of the prepared polymers were low (<10 kDa). Importantly, they observed that the methacrylates containing azobenzene groups connected in this way were easier to polymerize [16]. Nevertheless, a very long time was required to achieve higher conversion (192 h to achieve conversion 80% for one of the most active monomers among studied). It is noteworthy that such azobenzene polymethacrylates exhibited strong nonlinear optical properties [16].

Angiolini and coworkers [17] employed 1,1,4,7,10,10-hexamethyltriethylenetetramine (HMTETA) as a ligand in an ATRP catalytic system and allyl 2-bromoisobutyrate as an initiator in the synthesis of poly(meth)acrylates containing side-chain AZ groups. The azobenzene groups in the synthesized polymers were connected with a chain via an alkoxyl linker built of six carbon atoms. Additionally, azobenzene groups were substituted in para position by an alkoxyl chain with carbon atoms ranging from 2 to 24. The polymerization processes were carried out in dry tetrahydrofuran (THF). The applied conditions allowed for obtaining polymers with narrow dispersity (*Đ* = 1.12–1.17) and average molecular weight in the range 4.6–14.8 kDa. The synthesized polymers exhibit liquid-crystalline behavior. The transformations of smectic to nematic and subsequently isotropic phase were observed during the heating by differential scanning calorimetry (DSC) and polarized optical microscopy (POM).

Similarly, azobenzene-containing poly(meth)acrylates with an alkoxyl linker between AZ and polymer chains built of ten carbon atoms exhibit liquid-crystalline behavior. In a recent report, Yoshida and coworkers showed light-controlled transformations of liquid-crystalline phases using DSC, POM and wide-angle X-ray diffraction (WAXS) [18]. Two smectic phases were observed below the glass transition temperature, i.e., a transition into each other during the irradiation of polarized light, and the isotropic state above 107 °C.

Liquid-crystalline properties of poly(meth)acrylates containing side-chain azobenzene moieties connected via an alkoxyl linker built of three to six carbon atoms were studied by Imrie and coworkers. These polymers were synthesized using conventional free radical polymerization [19].

Thus far, the liquid-crystalline behavior of poly(meth)acrylates containing AZ-groups was observed only when a linker between these groups and a polymer chain is long (at least three carbon atoms). To the best of our knowledge, liquid-crystalline properties for the azobenzene poly(meth)acrylates with the shorter linkers were not yet observed.

This manuscript reports the synthesis of a series of poly(meth)acrylates containing side-chain azobenzene groups connected with a polymer chain via a short linker built of only one carbon atom (methylene group). The polymers are prepared via the ATRP technique using several catalytic systems. The optimization of polymerization conditions allows for obtaining narrowly dispersed polymers with molecular weights in the range of 2.6–14.3 kDa. The synthesized polymers exhibit photosensitive properties and liquid-crystalline behavior. The fabricated narrowly dispersed poly(meth)acrylates were analyzed using chromatographic (SEC), spectroscopic (UV–VIS, NMR), thermal (TGA, DSC), microscopic (POM), and X-ray scattering (WAXS) methods. We report the liquid-crystalline behavior of side-chain poly(meth)acrylates containing azobenzene groups connected with a polymer chain via a methylene linker for the first time.

## 2. Materials and Methods

### 2.1. Materials

#### 2.1.1. Solvents

Anhydrous anisole (Sigma-Aldrich, Bangalore, India; 99.7%), anhydrous methanol (Sigma-Aldrich, Steinheim, Germany; 99.8%), *d*-chloroform (Deutero, Kastellaun, Germany; 99.8%), cyclohexane (Avantor, Gliwice, Poland; 99.0%), dichloromethane (Avantor, Gliwice, Poland; 99.8%), diethyl ether (Avantor, Gliwice, Poland; extra pure), *d_6_*-dimethyl sulfoxide (Deutero, Kastellaun, Germany; 99.8%), ethyl acetate (Avantor, Gliwice, Poland; 99.8%), petroleum ether (Avantor, Gliwice, Poland; pure), tetrahydrofuran (Avantor, Gliwice, Poland; 99.8%) were used as received. In addition, 1,4-Dioxane (Avantor, Gliwice, Poland; extra pure) was predried with sodium hydroxide for 24 h and was stored above molecular sieves (3 Å).

#### 2.1.2. Reagents

Acryloyl chloride (Sigma-Aldrich, Budapest, Hungary; 97%), alumina oxide (Sigma-Aldrich, Buchs, Switzerland; 98%, Brockmann activity I), 4-aminobenzyl alcohol (Apollo Scientific, Stockport, United Kingdom; 99%), anhydrous acetic acid (Avantor, Gliwice, Poland; 99.6%) anhydrous magnesium sulphate (Avantor, Gliwice, Poland; extra pure), aniline (Acros Organics, Lizbona, Portugal; > 99%), ethyl 2-bromo-2-methylpropionate (EBriB, Sigma-Aldrich, Overijse, Belgium; 98%), ethyl 2-chloropropionate (ECP, Sigma-Aldrich, Chengdu, China; 97%), methacryloyl chloride (Sigma-Aldrich, Taufkirchen, Germany; 97%), *N*,*N*,*N*′,*N*″,*N*″-pentamethyldiethylenetriamine (PMDTA, Merck, Taufkirchen, Germany; 98%), potassium peroxomonosulfate (Oxone, Acros Organics, Morris Plains, NY, USA; extra pure, min. 4.5% active oxygen), sodium bicarbonate (Avantor, Gliwice, Poland; extra pure), sodium chloride (Avantor, Gliwice, Poland; extra pure), sodium hydroxide (Avantor, Gliwice, Poland; extra pure), triethylamine (Avantor, Gliwice, Poland; >99%), tris[2-(dimethylamino)ethyl]amine (Me_6_TREN, Sigma-Aldrich, Taufkirchen, Germany; 99%) were used as received. Copper(I) chloride (Sigma-Aldrich, Shanghai, China; 97%) and copper(I) bromide (Sigma-Aldrich, Shanghai, China; 97%) were rinsed with an aqueous solution of SO_2_, followed by a glacial acetic acid, anhydrous methanol, and finally with diethyl ether and dried in a vacuum oven at 80 °C for 3 days and stored under argon.

### 2.2. Methods

#### 2.2.1. NMR Spectroscopy 

Typical ^1^H and ^13^C spectra were recorded on a Bruker Avance III HD (^1^H: 300 MHz, ^13^C 100 MHz) or a Bruker Avance III HD (^1^H: 500 MHz) spectrometers at room temperature using *d*-chloroform or *d_6_*-dimethyl sulfoxide as solvents. NMR irradiation experiments were performed on Varian Mercury Plus (^1^H: 400 MHz) at room temperature using CDCl_3_ as a solvent, with Thorlabs M365F1 (365 nm, 4.1 mW) and M455F1 (455 nm, 24.5 mW) LEDs coupled to a 0.6 mm optical fiber, which was introduced to the NMR tube inside the spectrometer. ^1^H and ^13^C NMR chemical shifts were reported in δ units (ppm) relative to the residual solvent signal of CDCl_3_ (^1^H NMR, δ 7.26 ppm; ^13^C NMR, δ 77.0 ppm) or *d_6_*-DMSO (^1^H NMR, δ 2.50 ppm; ^13^C NMR, δ 39.5 ppm). The splitting pattern of peaks was designated as follows: s (singlet), d (doublet), t (triplet), p (quintet), m (multiplet).

#### 2.2.2. UV–VIS Spectroscopy

UV–VIS absorption spectra of the photoisomerization were measured on a Hewlett-Packard 8453 diode array spectrometer with Thorlabs M365F1 (365 nm, 4.1 mW) and M455F1 (455 nm, 24.5 mW) LEDs at room temperature using a 1 cm quartz cuvette. We used 1,4-dioxane (spectroscopic grade) as a solvent. The concentration of all samples was about 10 mg∙L^−1^. Kinetic measurements of thermal isomerization were measured using the above equipment. Samples were irradiated at 365 nm until no further changes were observed in the UV–VIS absorption spectrum, meaning that they reached the photostationary state. The changes of absorption at selected wavelengths were followed in time. The first-order decay rate constants at six temperatures were obtained by fitting to the equation f(x) = A∙exp(−t/k) + B using Origin software and Eyring plot analysis.

#### 2.2.3. Thermogravimetric Analysis (TGA) and Differential Scanning Calorimetry (DSC)

For the TGA, analyses were performed using TA Instruments TGA Q20 thermobalance under nitrogen atmosphere (flow rate of 40 mL∙min^−1^). The samples with a mass of ca. 6 mg were heated from room temperature to 800 °C at the heating rate of 20 K∙min^−1^.

For the DSC, measurements were performed using TA Instruments DSC Q20 calorimeter. A solid polymer sample with a mass of ca. 8 mg was heated in an aluminum pan from −20 °C to 180 °C at the rate of 20 K∙min^−1^. The glass transition temperature (*T_g_*) was determined to be the temperature that corresponded to half of the complete change in heat capacity in the second cooling cycle of the DSC scan. Measurements of thermal isomerization were carried out in an aluminum pan. First, a polymer sample was dissolved in dichloromethane and irradiated by an LED UV lamp (365 nm) until no further changes were observed in the UV–VIS absorption spectrum. Afterwards, the solvent was removed using a vacuum rotary evaporator at room temperature in the dark. The value of enthalpy of thermal isomerization was determined in the first heating cycle of the DSC scan. All the DSC measurements were performed under a nitrogen atmosphere (flow rate of 40 mL∙min^−1^).

#### 2.2.4. Size-Exclusion Chromatography (SEC) and Mass Spectrometry (HR-MS)

The analyses of average molecular masses of polymers were carried out using Shimadzu Nexera’s high-pressure liquid chromatograph equipped with a RID detector (Shimadzu RID–20A). The samples’ separations were performed in tetrahydrofuran (THF) using two columns placed in series: Reprogel 1000 and 10000 GPC, 5 μm, 300 mm × 8 mm. The columns were thermostated at 40 °C. GPC grade THF was used at a flow rate of 1.0 mL∙min^−1^. The concentration of samples prepared in the eluent was 1 mg∙mL^−1^; 20 μL injections were applied. Poly(styrene) standards were used for calibration (PSS Polymer) from 1 to 500 kDa.

High-resolution mass spectrometry was performed on a Shimadzu IT-ToF-MS spectrometer with electrospray ion source ionization using direct injection. The samples with mass 1 mg were dissolved in 1 mL of solvent (water/acetonitrile).

#### 2.2.5. Polarized Optical Microscopy (POM) and Wide Angle X-ray Scattering (WAXS)

Temperature-resolved microscopy was performed with an Imager A2m Zeiss polarizing microscope equipped with a Linkam heating stage. The samples were placed between two cover glass slips and gently sheared during the initial cooling from 140 °C. The intensity of the transmitted light was recorded on heating at 5 K∙min^−1^ with a shearing axis oriented at 45° with respect to the crossed polarizers.

Scattering patterns were collected with a Bruker D8 GADDS system (CuKα lamp, Goebel mirror point beam collimation) equipped with a Vantec2000 area detector. Polymer samples were deposited as droplets on a surface heated to 140 °C and cooled down to room temperature at 2 K∙min^−1^ prior to measurements. Silver behenate and silicon powder were used as low- and high-angle calibration standards, respectively.

#### 2.2.6. Theoretical Calculations 

All calculations were performed with the Gaussian 09 (RevD.01) software [20]. The optimization of geometry and vibrational frequencies were performed using the density functional theory (DFT) with Becke’s three-parameter hybrid functional and the functional correlation given by Lee et al. (DFT B3LYP/6-31G(d,p)). This is the same hybrid functional we successfully used earlier for DFT calculations of polymer systems [21,22,23,24]. The theoretical UV–VIS spectra were calculated using the time-dependent density functional theory (TD-DFT). All calculations were performed using the SMD solvation model [25] with parameters given for 1,4-dioxane.

### 2.3. Synthetic Procedures

#### 2.3.1. Synthesis of Nitrosobenzene (PhNO) 

Aniline (1.0 eq., 59 mmol, 5.4 mL) was dissolved in 170 mL of dichloromethane. Potassium peroxomonosulfate (Oxone, 1.0 eq., 59 mmol, 36 g) in water (180 mL) was added dropwise into the mixture. The reaction mixture was stirred vigorously at room temperature for about 0.5 h. Afterwards, the organic layer was separated from the aqueous phase. The last one was extracted with dichloromethane (3 × 50 mL), dried with anhydrous sodium sulfate, filtrated and concentrated using a rotary vacuum evaporator. The crude product was obtained as a dark-green oil (3.8 g, yield 60%) and used in the subsequent synthesis step without further purification.

#### 2.3.2. Synthesis of (4-(2-Phenyldiazen-1-yl)phenyl)methanol (AzoOH)

First, 4-aminobenzyl alcohol (0.9 eq., 32 mmol, 4.0 g) was dissolved in 150 mL of glacial acetic acid. Subsequently, the nitrosobenzene prepared in the first step (1.0 eq., 35 mmol, 3.8 g) was added, and the reaction mixture was stirred vigorously overnight at room temperature. Afterwards, the solvent was removed using a rotary vacuum evaporator. The solid product was dissolved in 200 mL of ethyl acetate, washed with a saturated aqueous sodium bicarbonate solution (3 × 60 mL) and brine (2 × 40 mL). The organic layer was separated and dried over anhydrous magnesium sulfate, filtrated and concentrated using a rotary vacuum evaporator. The crude product was purified by normal phase column chromatography (silica gel, gradient elution petroleum ether/ethyl acetate from 4:1 to 2:1). The product was obtained in the form of an orange solid (4.0 g, yield 59%). ^1^H NMR (300 MHz, *d_6_*-DMSO) δ 7.99–7.85 (m, 4H), 7.59–7.41 (m, 5H), 4.79 (d, J = 2.6 Hz, 2H), 1.85 (s, 1H); ^13^C NMR (101 MHz, *d_6_*-DMSO) δ 151.95, 150.83, 146.48, 131.35, 129.45, 127.12, 122.46, 122.44, 62.44; HR-MS (ESI): [M+H]+ 213.0931 (found), 213.1022 (calculated); UV–VIS (MeOH): λ_max_ = 322 nm; m.p.: 138–139 °C.

#### 2.3.3. Synthesis of (4-(2-Phenyldiazen-1-yl)phenyl)methylprop-2-enoate (AzoAA)

AzoOH (1.0 eq., 17 mmol, 3.5 g) was dissolved in 100 mL of dichloromethane, and triethylamine (2.0 eq., 18 mmol, 4.6 mL) was added. The solution was stirred for 20 min. Then, acryloyl chloride (1.1 eq., 18 mmol, 1.5 mL) was added dropwise, and the reaction mixture was stirred overnight at room temperature. The reaction mixture was washed with water (3 × 30 mL), 10% aqueous solution of hydrochloric acid (3 × 30 mL), 1 M aqueous solution of sodium hydroxide (3 × 30 mL), and brine (2 × 30 mL). The organic phase was separated, dried with anhydrous magnesium sulfate, filtrated and concentrated using a rotary vacuum evaporator. The crude product was purified by normal phase column chromatography (silica gel, cyclohexane/ethyl acetate = 20:1). The product was obtained in the form of an orange solid (3.5 g, yield 80%). ^1^H NMR (300 MHz, CDCl_3_) δ 8.00–7.89 (m, 4H), 7.61–7.44 (m, 5H), 6.51 (dd, J = 17.3, 1.5 Hz, 1H), 6.23 (dd, J = 17.3, 10.4 Hz, 1H), 5.91 (dd, J = 10.4, 1.5 Hz, 1H), 5.31 (s, 2H); ^13^C NMR (75 MHz, CDCl_3_) δ 166.07, 152.76, 152.56, 138.86, 131.53, 131.28, 129.25, 128.90, 128.31, 123.20, 123.04, 65.90. HR-MS (ESI): [M+H]+ 267.0971 (found), 267.1128 (calculated); UV–VIS (1,4-dioxane): λ_max_ = 323 nm; m.p.: 75–77°C.

#### 2.3.4. Synthesis of (4-(2-Phenyldiazen-1-yl)phenyl)methyl-2-methylprop-2-enoate0, (AzoMA)

AzoOH (1.0 eq., 19 mmol, 4.0 g) was dissolved in 100 mL of dichloromethane, and triethylamine (2.0 eq., 28 mmol, 5.2 mL) was added. The solution was stirred for 20 min. Then, methacryloyl chloride (1.1 eq., 20 mmol, 2.0 mL) was added dropwise, and the reaction mixture was stirred overnight at room temperature. Reaction mixture was washed with water (3 × 30 mL), 10% aqueous solution of hydrochloric acid (3 × 30 mL), 1 M aqueous solution of sodium hydroxide (3 × 30 mL), and brine (2 × 30 mL). The organic phase was dried over anhydrous magnesium sulfate, filtrated and concentrated using a rotary vacuum evaporator. The crude product was purified by normal phase column chromatography (silica gel, cyclohexane/ethyl acetate = 20:1). The product was obtained in the form of an orange solid (4.7 g, yield 90%). ^1^H NMR (400 MHz, CDCl_3_) δ 7.96–7.88 (m, 4H), 7.54–7.44 (m, 5H), 6.20 (t, J = 1.3 Hz, 1H), 5.62 (p, J = 1.6 Hz, 1H), 5.28 (s, 2H), 2.00 (t, J = 1.3 Hz, 3H); ^13^C NMR (101 MHz, CDCl_3_) δ 167.3, 152.8, 152.5, 139.1, 136.3, 131.3, 129.2, 128.7, 126.2, 123.2, 123.0, 66.0, 18.5; HR-MS (ESI): [M+H]+ 281,1288 (found), 281,1284 (calculated); UV–VIS (1,4-dioxane): λ_max_ = 324 nm; m.p.: 84–85°C.

#### 2.3.5. Polymerization of AzoAA and AzoMA

All polymerizations were performed using the ATRP technique. Several catalyst systems under various conditions were applied. The molar ratios, reagents, and conditions of polymerization are given in Table 1. Briefly, in a typical experiment, the monomer (AzoAA or AzoMA) and catalyst (CuCl or CuBr) were added to a dried Schlenk flask sealed with a silicone rubber septum and equipped with a magnetic stir bar. The flask was degassed using a Schlenk line and filled with dry argon. Next, a dry solvent (1,4-dioxane or anisole) was added to the flask. The polymerization mixture was bubbled with argon for 0.5 h. Next, an appropriate ligand volume (PMDTA or Me_6_TREN) was injected into the flask using a syringe in argon’s counterflow. The mixture was stirred and bubbled with argon for the next 30 min. After this time, an initiator (ECP, EBriB) was injected into the flask using a syringe in argon’s counterflow. The mixture was immersed in an oil bath on a magnetic stirrer at an appropriate temperature (65, 95 or 120 °C) for a proper time duration (from 24 to 144 h). The polymerization was terminated by blowing air through the flask. The post-polymerization mixture was viscous, dark orange, and homogeneous. Subsequently, the polymerization mixture was dissolved in THF, concentrated using a rotary vacuum evaporator, and passed through an alumina column to remove the catalyst system. The polymers were purified by precipitation with cold methanol. Lastly, the solid products were dried in a vacuum oven for 48 h at 45 °C. The monomer conversion was determined gravimetrically, while the average molecular masses were obtained by SEC. ^1^H NMR spectra proved that the purified polymers do not contain any residuals of monomer (see Figure S1 in Supplementary Materials).

## 3. Results and Discussion

Two azobenzene derivatives of acrylic and methacrylic acid were synthesized and were used to prepare narrow-dispersive PRPs. Properties of the synthesized polymers, with particular emphasis on their photochemical and crystalline behavior, were studied in detail.

**Scheme 1:**
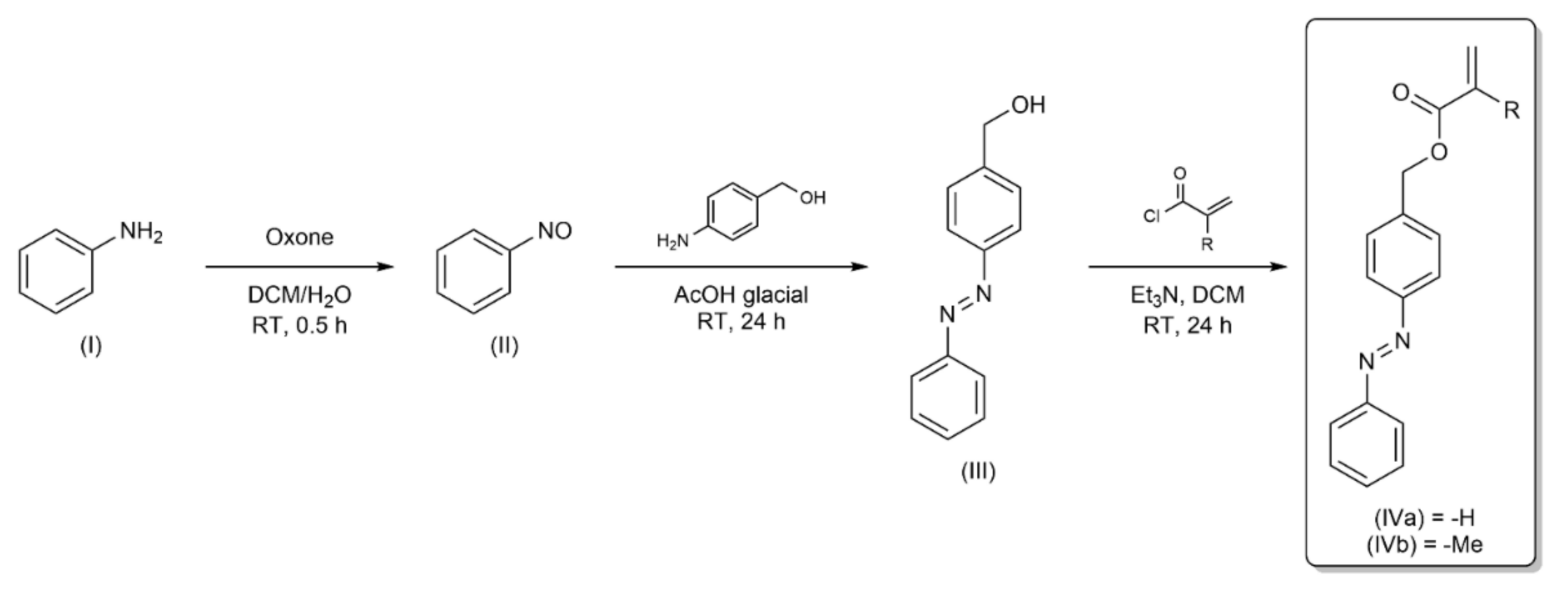
The route of syntheses of photosensitive (meth)acrylate monomers.

The developed syntheses of azobenzene-monomers consisted of three stages (Scheme 1). First, nitrosobenzene (II) was obtained in heterophasic oxidation of aniline (I) by Oxone (Caro’s acid salt). In the second step, we obtained an azobenzene derivative containing CH_2_–OH (AzoOH, III) moiety by coupling the 4-aminobenzyl alcohol and nitrosobenzene (II) via the Mill’s reaction. In the last step, we carried out an esterification reaction between AzoOH (III) and appropriate acyl chloride (acryloyl chloride or methacryloyl chloride), in turn receiving two photosensitive monomers: AzoAA (IVa) and AzoMA (IVb).

**Figure 1:**
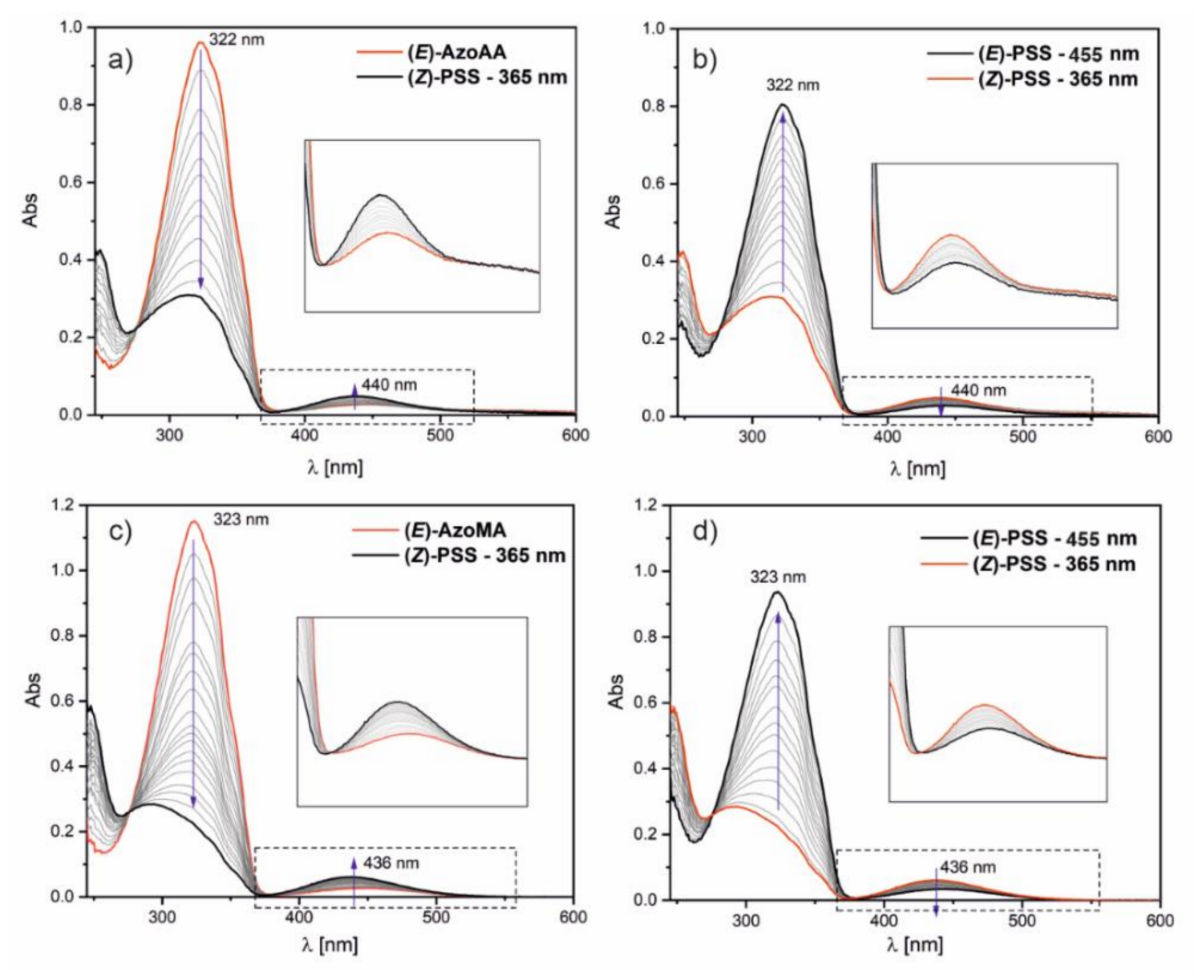
UV–VIS absorption spectra of AzoAA (**a**), (**b**) and AzoMA (**c**), (**d**) recorded in solution (1,4-dioxane) under illumination at 365 nm: (**a**), (**c**); and at 455 nm: (**b**), (**d**). The spectra were recorded at 30 s intervals. The arrows indicate the time sequence of recorder spectra.

The photosensitivity of the synthesized monomers was analyzed using theoretical and experimental studies. UV–VIS spectroscopy allows us to follow the photoisomerization phenomenon of azobenzenes under illumination. Monomers’ solutions in 1,4-dioxane were irradiated by LEDs (365 and 455 nm) at room temperature (without cooling) over 15 min. The monomer’s solution was illuminated first at 365 nm, and UV–VIS absorption spectra were recorded every 30 s. When the photostationary state (PSS) was reached, illumination was continued using light with wavelength 455 nm until the next PSS was established. Figure 1 displays the changes in absorbance in the UV–VIS range under illumination at 365 nm and 455 nm.

In the UV–VIS spectra of AzoAA and AzoMA, two bands are observed: the strong band with a maximum at 322 nm for AzoAA and 323 nm for AzoMA. The band corresponds to π–π^*^ electron transition; the band with a maximum at 440 nm for AzoAA and 436 nm for AzoMA is very weak and corresponds to n–π^*^ transition. Such a position of the bands indicates the trans structure of the azobenzene group in the molecules [26,27]. During the irradiation with 365 nm light, the first band’s intensity gradually decreased, and the second band intensity gradually increased (see Figure 1a,c). It is a consequence of the photoisomerization trans-cis (*E*→*Z*) process. As shown in Figure 1, the process of photoisomerization is entirely reversible for the synthesized monomers. The trans-cis transformation (*Z*→*E*) of the compounds occurred when the solution was illuminated by light in the visible range (Figure 1b,d).

**Figure 2:**
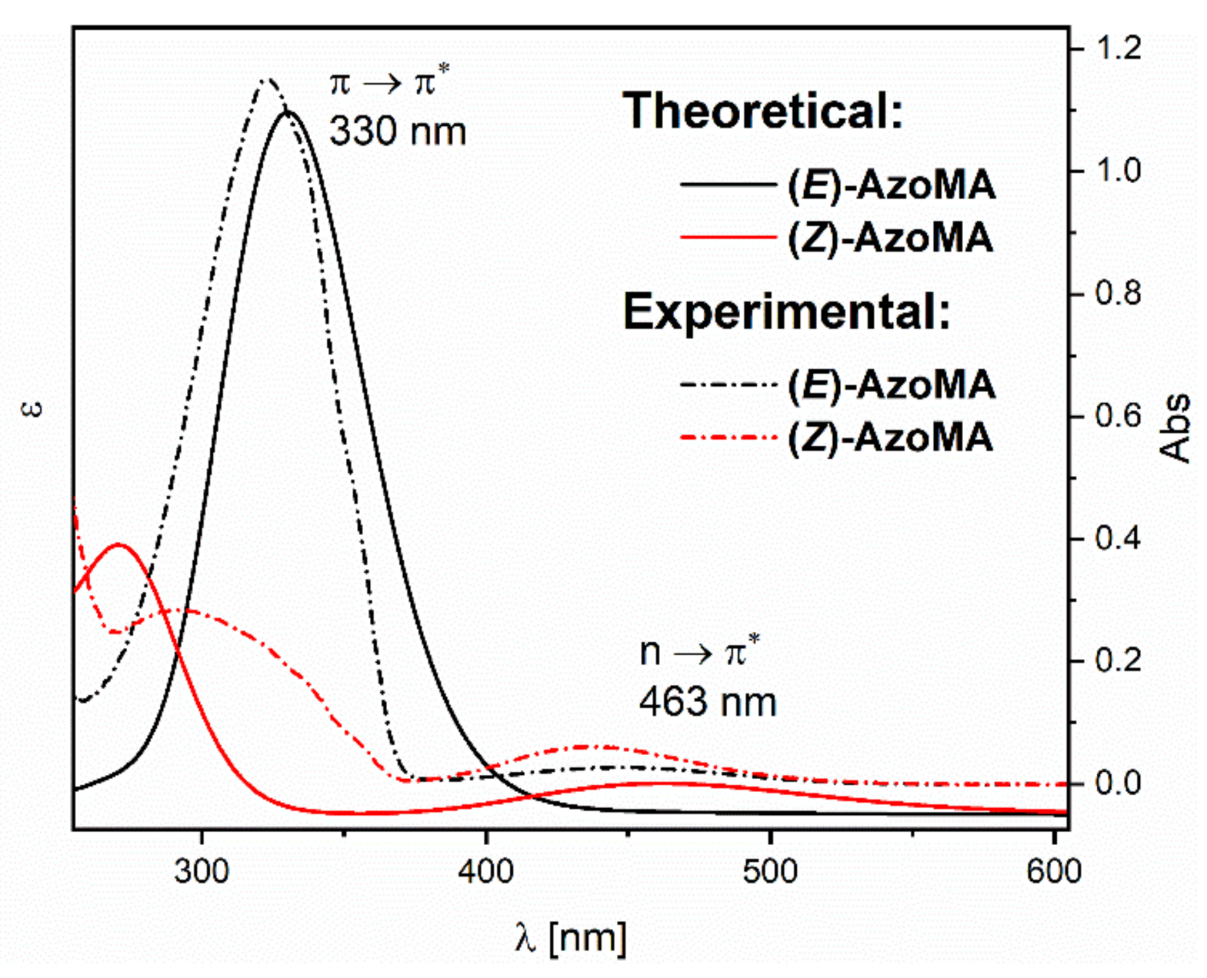
Theoretical UV–VIS spectra with assigned maxima (solid line) determined using TD-DFT CAM-B3LYP/6-31+G(d,p) method compared with experimental UV–VIS spectra (dotted line) for (*E*)-AzoMA and (*Z*)-AzoMA.

The experimental UV–VIS spectra recorded for the AzoMA were compared with the theoretical spectra derived from quantum-mechanical calculations using time-dependent density functional theory (TD-DFT) with hybrid functional CAM-B3LYP with a standard orbital basis set implementation (TD-DFT CAM-B3LYP/6-31+G(d,p)). In the calculations, the solvation model density (SMD) continuum model [25] was used to consider the solvent molecules’ influence (in our case, 1,4-dioxane). Due to the practically identical course of experimental UV–VIS spectra for AzoAA and AzoMA (see Figure 1), theoretical spectra were determined only for one of them, namely AzoMA. Figure 2 shows the theoretical UV–VIS spectra for AzoMA isomers as theoretical extinction coefficient vs. wavelength. As seen in Figure 2, the trans isomer’s theoretical spectrum (black curve) has a strong band with a maximum at 330 nm, which corresponds to a π–π^*^ transfer. The theoretical spectrum has a characteristic band in a visible range with a maximum at 463 nm for the cis isomer. The slight differences in the bands’ position in experimental and theoretical spectra are probably a consequence of the chosen basis set (6-31+G(d,p)). It is most likely that the calculations using this basis set are not accurate enough to determine the interaction of AzoMA with the solvent’s molecules.

ATRP was used to polymerize the synthesized photosensitive monomers using several types of catalytic systems. A series of polymerizations was carried out by differing, besides a catalytic system, the temperature, solvent, polymerization time, and reagents’ molar ratios. The conditions of the polymerizations are presented in Table 1.

The polymerizations of AzoAA (1A to 6A) were carried out in 1,4-dioxane at 65 °C and 95 °C using *N*,*N*,*N*′,*N*″,*N*″-pentamethyldiethylenetriamine (PMDTA) as a ligand in the catalytic system. The ethyl 2-chloropropionate (ECP) was used as an initiator in these systems, while CuCl was used as a catalyst. If the catalytic system contained CuBr, then the ethyl 2-bromoisobutyrate (EBriB) was applied as an initiator. In such a way, we tested two types of catalytic systems differing in activity since the chlorine atom from a catalytic complex combines with growing polymer macroradicals stronger than bromine, and polymerizations run slower. We did not obtain polymers when polymerizations of AzoAA were carried out at 65 °C using PMDTA/CuCl/ECP system, even for a long polymerization time (96 h). The application of EBriB as an initiator and CuBr/PMDTA as a catalytic system enabled polymerization. However, the achieved conversions and molecular weights of the obtained poly(AzoAA) are relatively low (see Table 2). As it can be expected, AzoMA exhibited higher activity in polymerization than AzoAA. We tested two catalytic systems, namely CuBr/PMDTA and CuBr/Me_6_TREN. In all reactions, the EBriB was used as an initiator. First attempts were performed in 1,4-dioxane at 95 °C for two molar monomer ratios to the initiator (50:1 and 100:1). The time of polymerization was in the range of 24–144 h.

In Table 2, the synthesized polymers’ average molecular weights *M_n_* (from SEC analyses) and achieved monomer conversions are presented. The experimental molecular weights are compared with theoretical molecular weights calculated based on the molar ratio of reagents and monomer conversion (*α*). We tried to determine the molecular masses of the synthesized polymers from ^1^H NMR spectra analogically to the approach by Kostjuk and coworkers [28]. Unfortunately, in the ^1^H spectra of the synthesized polymers, the signals of protons from the end groups (methyl and ethyl protons from the initiator moiety) overlap with protons’ signals from the main chain methyl and methylene protons (see Figure S1 in Supplementary Materials).

As a result of the polymerizations of AzoAA performed according to the description in positions 1A–3A in Table 1, we did not obtain polymers. The extension of the polymerization time to 96 h allowed us to obtain poly(AzoAA) but only with a low yield (conversion < 5%). We had a problem with the polymer’s isolation from the post-polymerization mixture; this is ascribable to its very low mass. The determined polydispersity index for the synthesized polymer is low (1.15), which indicates that the process was well-controlled and that the termination reactions were limited.

To achieve a higher yield of poly(AzoAA) synthesis in a shorter time, we decided to increase the temperature and change a catalytic system to CuBr/PMDTA/EBriB, which was reported to give faster rates of ATRP polymerization. After 72 h of polymerization using the CuBr/PMDTA/EBriB system, the conversion of AzoAA was 20%, and the molecular mass *M_n_* determined from SEC was 2.6 kDa. However, the dispersity of molecular mass was not very narrow (Đ = 1.37).

The same catalytic system was employed in the polymerization of AzoMA for two molar ratios of reagents (see Table 1). The polymerization reactions, denoted in Table 1 as 1M to 6M, using a 50:1:1:1 molar ratio, differ in time ranging from 24 h to 132 h. In Table 2, the results of SEC analyses for the obtained polymers are presented. With increasing polymerization time, the molecular weights of the synthesized polymers and conversion of monomer increased. The molecular masses of the polymers are in a range of 4.7 kDa to 10.7 kDa.

**Figure 3:**
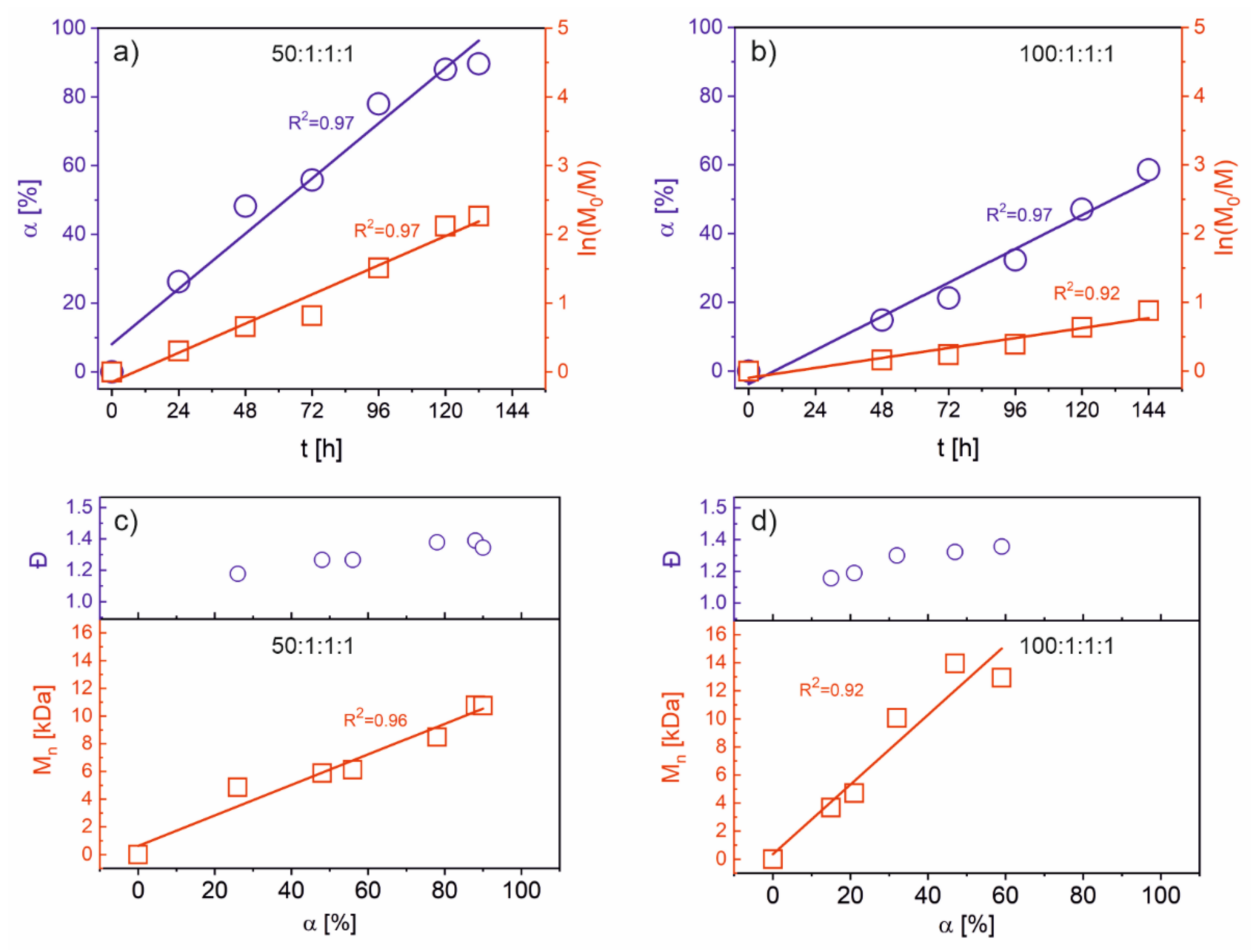
The conversion of monomer (α) vs. time (the blue line), first-order kinetic curve (the red line) (in (**a**) and (**b**)), the dependence of number-average molecular weight (the red points), and molecular weight distribution (the blue circles) (in (**c**) and (**d**)) determined for the polymerization of AzoMA performed under conditions according to the description in Table 1 (1M–11M).

Figure 3a illustrates the relationship between monomer conversion vs. time and the kinetic curve for the polymerization of AzoMA (1M–6M in Table 1). The determined molecular masses and polydispersity indexes determined from SEC analyses for the prepared polymers are presented in Figure 3c.

As shown in Figure 3a, the conversion of AzoMA increased linearly with time, and the correlation is considered good (R^2^ = 0.97). The reaction’s kinetics can be described as a first-order reaction with respect to the monomer concentration with fairly good approximation (R^2^ = 0.97). The molecular masses of the prepared polymers increased with time; the relationship is linear with a relatively good correlation (R^2^ = 0.96); and the Đ values for the polymers are below 1.4 for all the polymers (see Figure 3c). This indicates that the polymerization of AzoMA using CuBr/PMDTA as a catalytic system performed at 95 °C in dioxane was well controlled, and the termination processes were significantly limited. In addition, compared with the results reported by Lu and coworkers [15] for a similar system, under our conditions, a substantially shorter time of polymerization was necessary to achieve high conversion. This indicates that the monomers designed by us were more reactive than those prepared by Lu and coworkers [15]. The conditions of the polymerizations allowed for faster kinetics with a similar degree of control.

We tested the same catalytic system with a higher molar ratio of the monomer to the initiator (100:1). Interestingly, under the same conditions, significantly smaller monomer conversions were recorded for the corresponding times of polymerization. The polymerization ran slower, and the dispersity of molecular masses was narrower (Đ < 1.3). The observed system decreased in the polymerization rate; this was caused by the reduction of the initiator and catalyst concentration, which can be on the basis of the kinetic equation describing the propagation process in the ATRP polymerization.

The solvent change in the polymerization system from 1,4-dioxane to anisole allowed us to perform this process at a higher temperature (120 °C). CuBr was used as a catalyst with two types of ligands: PMDTA and Me_6_TREN. When the last ligand was employed, the polymerization proceeded very quickly. After 28 h, conversion is 54%, and after 48 h, the polymerization was practically completed (conversion 90%). However, the process was weakly controlled by the catalytic system, and the dispersity of molecular masses in the synthesized materials was high (*Đ* = 1.59 and 1.60, after 28 h and 48 h, respectively). This shows that the application of Me_6_TREN in the polymerization of azobenzene-methacrylates gives a weak control. In contrast, the polymerization of AzoMA using CuBr as a catalyst and PMDETA as a ligand in anisole at 120 °C (12M in Table 1) ran relatively fast (conversion 32% after 24 h). However, the polydispersity of the synthesized polymer was high (*Đ* = 1.66).

In summary, the best results were achieved for the polymerizations of AzoMA using the CuBr/PMDETA/EBriB system, performed in 1,4-dioxane. The applied conditions allowed us to obtain polymers with a relatively narrow dispersity (*Đ* < 1.35).

**Figure 4:**
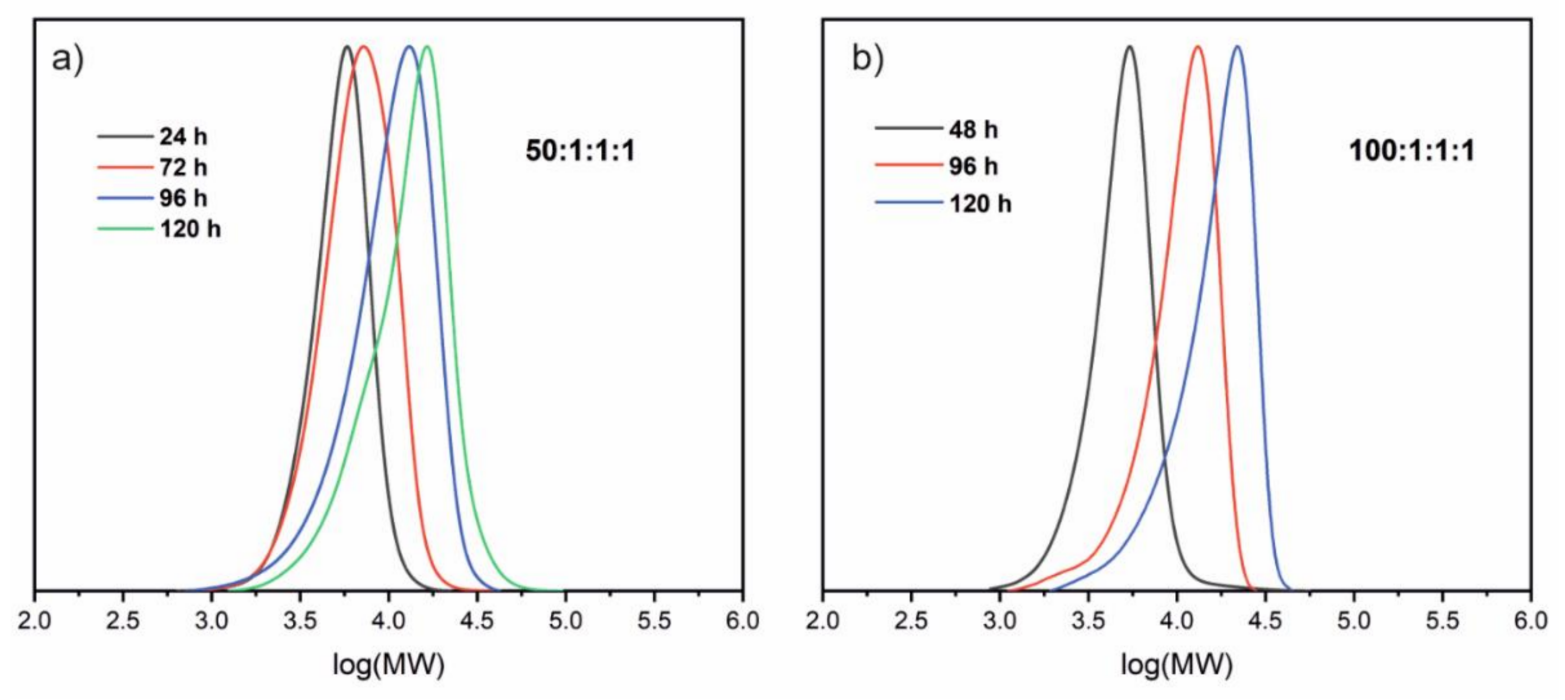
The MWD curves obtained from SEC analyses for poly(AzoMA) for two molar monomer: initiator ratios (50:1 (**a**) and 100:1 (**b**)); the polymers were prepared according to the description in Table 1.

Figure 4 shows molecular weights distribution (MWD) for the selected synthesized polymers obtained from SEC analyses. The curves illustrating MWD for all prepared polymers are presented in the Supplementary Materials (Figure S2). Generally, for a short polymerization time (24–72 h) for both molar ratios, molecular masses distributions are relatively narrow, and MWD curves are symmetric. MWD curves become wider for longer times with the tailing effect due to the termination processes such as recombination or chain transfer reactions (see Figure S2 in Supplementary Materials).

The thermal stability of fabricated polymers was examined by thermogravimetric analysis (TGA). The synthesized photoresponsive polymers exhibited high thermal stability, significantly higher than the monomers. Figure S3 in the Supplementary Materials shows TGA curves recorded for the selected polymers during the heating from room temperature to 800 °C. The thermal decomposition of the polymers began at 300 °C, only with one exception—sample 1M, whose decomposition was 100 degrees higher than that in the case of monomers. The high thermal stability of the synthesized polymers is crucial for their potential applications and enables the typical processing of thermoplastic melts. The lower stability that was observed for sample 1M corresponds well with the smallest molecular weight among the studied.

**Figure 5:**
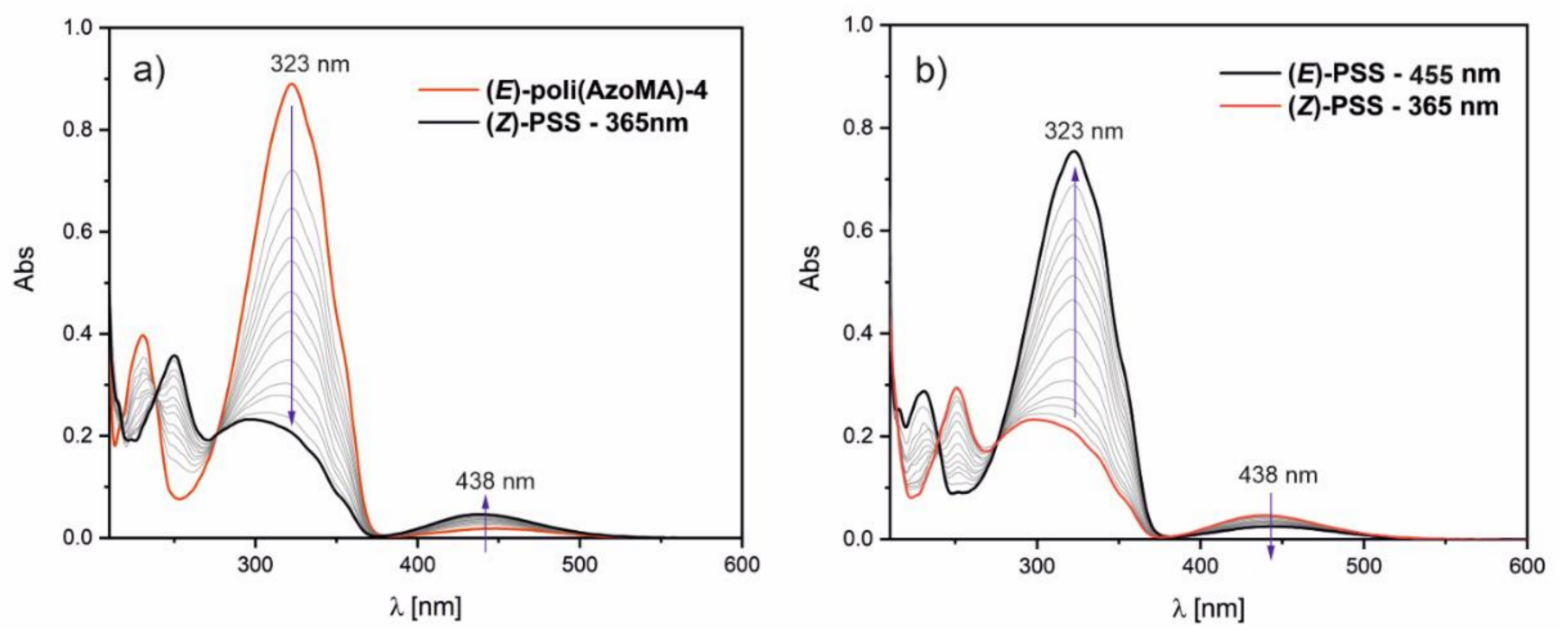
UV–VIS absorption spectra of poly(AzoMA) (Sample 4M) recorded in solution (THF) under illumination with 365 nm (**a**) and 455 nm (**b**) light.

The synthesized polymers’ photochemical behavior was studied using UV–VIS and NMR spectroscopy. Figure 5 displays UV–VIS spectra of the selected sample of the synthesized poly(AzoMA) recorded in THF solution under an illumination of 365 nm (Figure 5a) and 455 nm (Figure 5b). The observed spectra are practically identical to those registered for the polymer’s monomer (see Figure 1c,d). The isosbestic points at 240, 274, and 377 nm in these spectra prove a lack of degradation of azobenzene groups during long illumination. Furthermore, the similarity of monomer’s and polymer’s spectra indicates that the transformations of azobenzene groups in the polymer chain occur independently.

**Figure 6:**
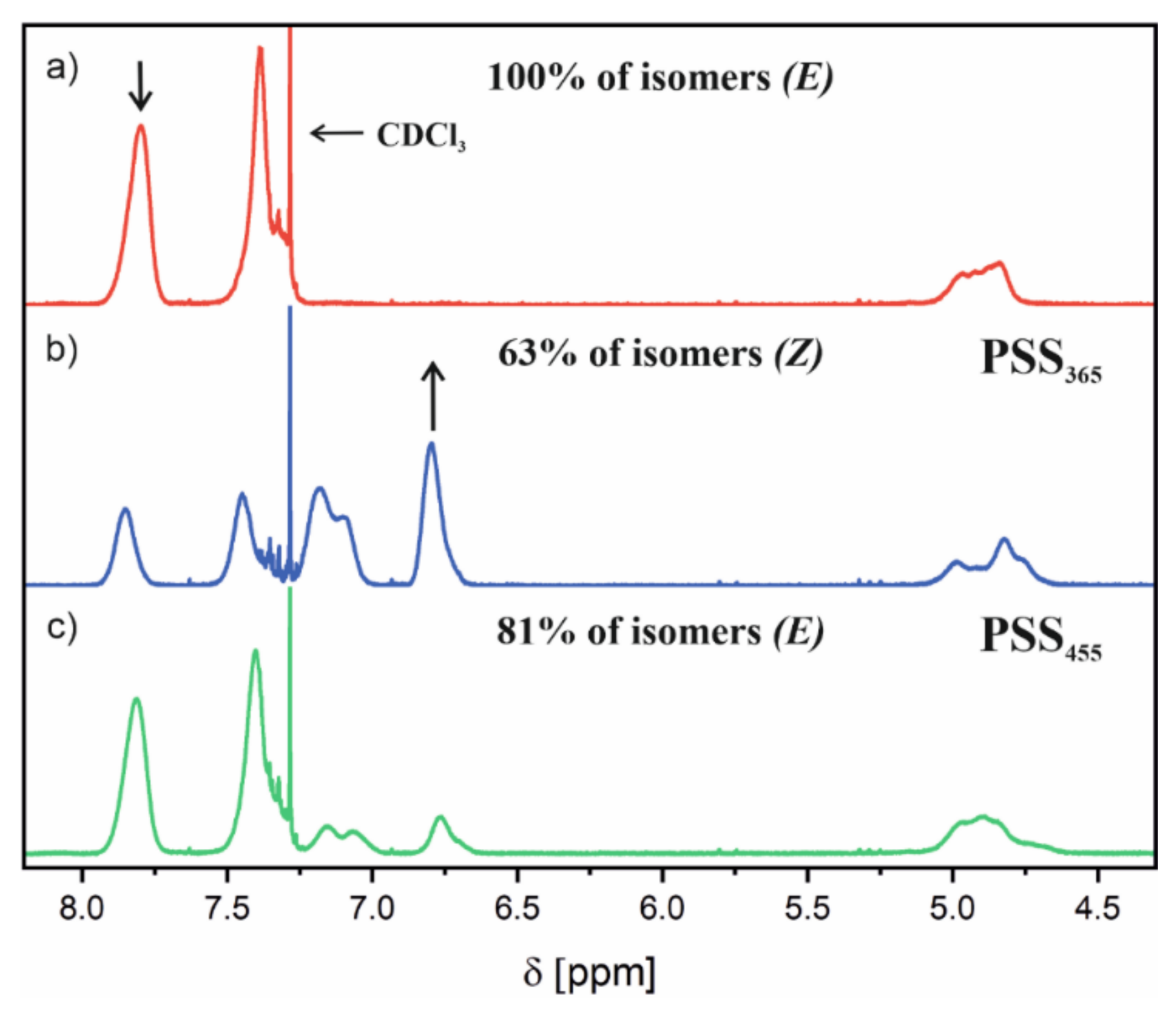
^1^H NMR spectra of poly(AzoMA) recorded without illumination (**a**); after irradiation with 365 nm light (after reaching the photostationary state—PSS_365_) (**b**); after subsequent exposition to 455 nm light (after reaching the photostationary state—PSS_455_) (**c**).

^1^H NMR spectroscopy allowed us to explore the isomerization of the synthesized polymers quantitatively. To achieve this, we used a spectrometer with a specially constructed illumination system (see Figure S4 in Supplementary Materials). Figure 6 shows the ^1^H NMR spectra recorded for the selected poly(AzoMA) which was obtained according to the description in Table 1 (Sample 4M). The spectrum denoted in Figure 6a was recorded for the sample without light; the spectra in Figure 6b and in Figure 6c were recorded at the photostationary states after irradiation at 365 nm and 455 nm, respectively. Under the influence of light at 365 nm, the signal at 7.8 ppm—which corresponds to 4H in ortho positions relative to the azo group—was seen to decrease, while the signal at 6.8 ppm appears and continues to increase during the progress of isomerization. The reverse changes occur when the sample was subsequently illuminated with 465 nm light. The signal at 7.8 ppm is present in the trans isomer of the azobenzene group; as a result of the transformation to a cis isomer, this signal shifted to 6.8 ppm. We calculated the mutual quantity of both isomers in the sample from the relative integration of these signals. The estimated contribution of both isomers in the polymer sample is given in Figure 6.

We compared the observed changes in the ^1^H NMR spectrum of poly(AzoMA) under illumination with analogical changes for the monomer (see Figure S5 in Supplementary Materials). The differences are not significant, although the dynamics of the transformation of *E* to *Z* isomer are slower than in the inverse process. After the illumination with 365 nm in the photostationary state (PSS_365_), the cis isomer’s content is 75%, which is higher than that in the polymer (63%). However, the reverse transformation to the trans isomer affected by light at 455 nm led to a very similar composition of both isomers in the photostationary state (PSS_455_). It seems that the azobenzene groups undergo isomerizations independently, and the polymer chain does not influence these processes. However, the isomerizations of azobenzene groups may affect the motions of the polymer chain [29]. Such effects can be analyzed using differential scanning calorimetry.

**Figure 7:**
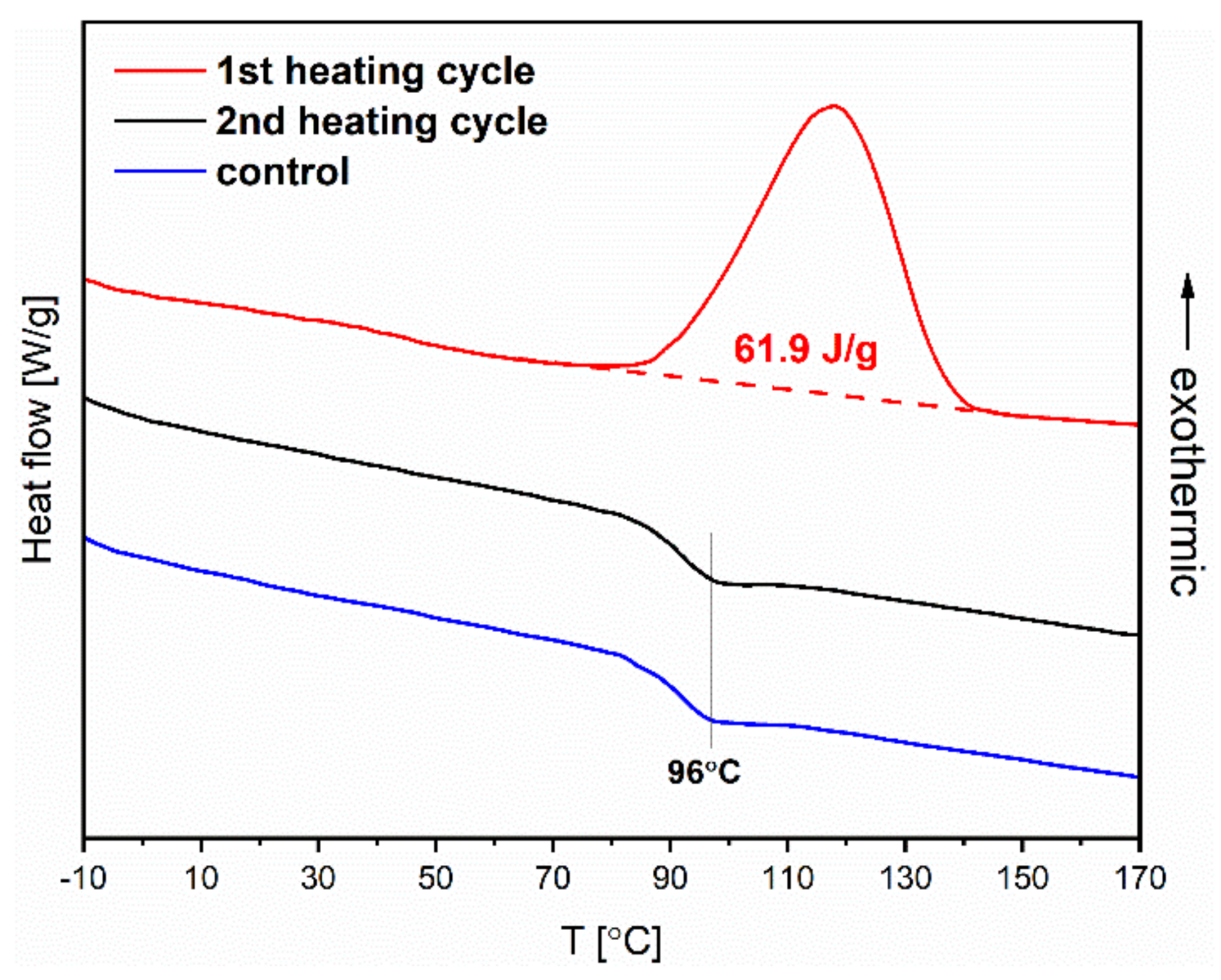
DSC thermograms recorded for the solid poly(AzoMA) in the first heating cycle (red curve) and in the second heating cycle (black curve) (Sample 4M). The sample was illuminated in 1,4-dioxane solution using 365 nm light to the photostationary state before solvent evaporation and the DSC run. The control curve (blue) was recorded for the sample, which was not illuminated earlier. The vertical line indicates glass transition.

For the one exemplified poly(AzoMA) sample (4M), the enthalpy of isomerization *Z*→*E* was determined using DSC. For this purpose, the sample was first dissolved in 1,4-dioxane and was illuminated at 365 nm until a photostationary state was achieved. Then, the solvent was evaporated, and the solid polymer was analyzed using DSC. Figure 7 shows the recorded DSC thermogram in the heating cycle (red curve). The exothermic peak corresponds to the thermal isomerization *Z*→*E* of azobenzene groups in a polymer chain. The process of releasing energy after earlier illumination enables the energy storage in such material. Grossman and coworkers [30] showed that polymethacrylates containing azobenzene groups could be potentially employed as solar energy capacitors for solid-state solar thermal fuels (STFs). The enthalpy of the isomerization *Z*→*E*, determined from DSC, for the fabricated polymer by us is relatively high at 61.9 J∙g^−1^ (17 Wh∙kg^−1^), which indicates its potential for energy storage applications. Considering this possibility of applying the fabricated materials, we decided to evaluate the kinetics of reverse spontaneous isomerization *Z*→*E* at room temperature (20 °C). The Eyring–Polanyi (E-P) equation was used to analyze the isomerization of AzoMA (for the exemplified polymer 4M). The UV–VIS measurements were performed at several temperatures, and absorbance changes in time for the selected wavelength were recorded. The determined linear relationships are presented in the Supplementary Materials (Figure S7). On the base of the Eyring plots, a half-life time (*τ_1/2_*) and a rate constant (*k*) of the isomerization at 20 °C were determined. The free energies of activation (Δ*G^‡^*(20 °C)) of the isomerization process for AzoMA and selected sample of poly(AzoMA) were also calculated (see Table S1 in the Supplementary Materials). The kinetic parameters calculated from the E-P equation show that the rate of *Z*→*E* isomerization for the analyzed polymer was relatively slow (*τ_1/2_* = 3.3 days) and was two times faster than for its monomer. In comparison with polymers tested by Grossman and coworkers, our materials characterize higher free energies of activation and longer half-life time. Notably, the relatively long half-life time (*τ_1/2_* = 3.3 days) is highly suitable for long-term energy storage in applications requiring daily cycles [30].

DSC analyses allowed us to determine the glass transition temperatures for the fabricated polymers. The determined values of *T_g_* for the selected samples of polymers are presented in Table 3, which also presents *M_n_* and *Đ* values for these polymers from SEC. The chosen polymers characterize the narrowest dispersity among the synthesized and represent the polymers’ whole range of molecular weights.

Figure S8 in the Supplementary Materials shows the exemplified thermogram from DSC analysis (for the sample 10M). Data were collected in the second heating–cooling cycle after the vitrification of the samples. The *T_g_* values were determined in a second cooling cycle. They correlate well with molecular weights; namely, the expected increase of *T_g_* with increasing *M_n_* is observed. However, an additional effect below *T_g_* was observed in a first heating cycle (not illustrated in Figure S8), indicating most probably phase transition. Therefore, we decided to investigate the internal structure of the prepared polymers using X-ray diffraction.

**Figure 8:**
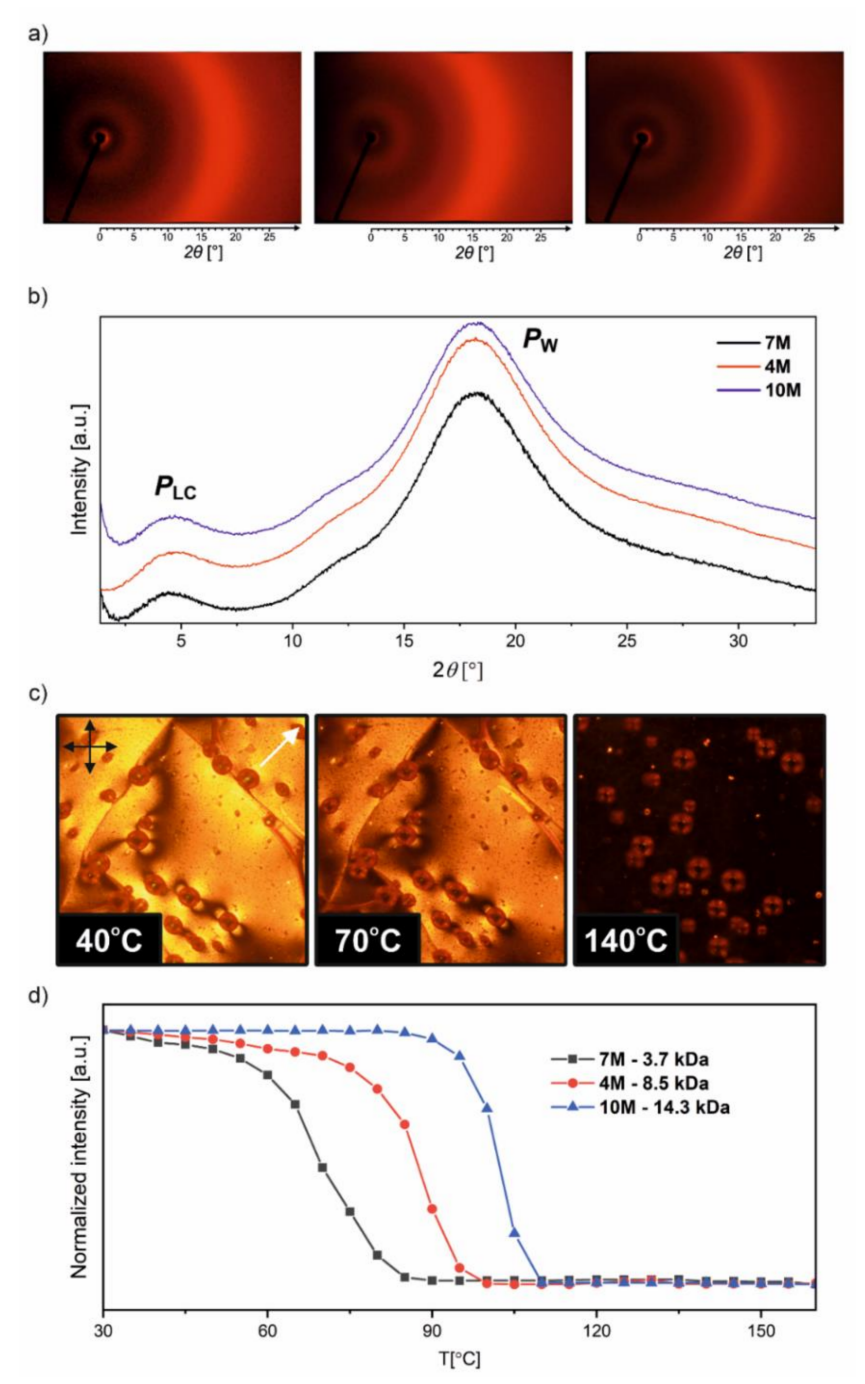
X-ray scattering patterns of poly(AzoMA) 7M, 4M, and 10M (from the left) collected at room temperature (**a**). Azimuthally-integrated data of 7M, 4M, and 10M. The profiles were shifted vertically for clarity (**b**). POM microscopic images between crossed polarizers of selected poly(AzoMA) (Sample 4M) at different temperatures (40, 70 and 140 °C). The white arrow indicates the direction of the shearing axis (**c**). Temperature dependence of the optical birefringence for selected poly(AzoMA)s (Samples 4M, 7M, and 10M) (**d**).

Room-temperature scattering patterns were collected over the small- and wide-angle range using a general area detector diffraction system (GADDS). In the wide-angle region, the patterns (Figure 8a,b) contained only a broad signal at ≈19° (2θ) corresponding to the side packing of the azobenzene units and the polymer backbone, indicative of the liquid-like structure and the lack of long-range crystalline order in these materials. Despite the short linker, the presence of a liquid-crystalline (LC) nematic (N) phase is indicated by a peak at ≈5° (2θ) in the small-angle region corresponding to a characteristic correlation spacing of 18.1 Å (7M), 18.9 Å (4M), and 19.2 Å (10M) between the mesogens in the homologue series (Table S2 in Supplementary Materials). The nematic order of the samples is also evidenced by the birefringent textures in thin films observed under a polarized light optical microscope (Figure 8c,d). The N–Iso phase transitions recorded on heating are broad and shift towards higher temperatures for higher molecular weight homologs ranging from 70 °C for 7M to 102 °C for 10M material (Table 3). We noted, however, that the N-Iso transitions were not clearly resolved in the DSC curves due to the narrow temperature window of the LC phase (especially in the higher MW phase homologues) caused by the vitrification and the overlap with the glass transition.

## 4. Conclusions

We reported the liquid-crystalline behavior of side-chain poly(meth)acrylates containing azobenzene groups connected with polymer chain via a short methylene linker for the first time. A series of side-chain azobenzene poly(meth)acrylates were synthesized via the ATRP technique using several catalytic systems. Among the studied systems, the one composed of CuBr and PMDETA proved to be optimal. The polymers with narrow molecular weight distribution (Đ ≤ 1.35) and the average molecular weights in the range of 2.6–14.3 kDa were synthesized. In comparison with earlier reports, the conditions of ATRP used in our syntheses allowed for obtaining relatively narrowly dispersed azobenzene-poly(meth)acrylates in a shorter time. The photoisomerization of azobenzene groups in the synthesized monomers and their polymers was analyzed using UV–VIS and NMR spectrometry. Theoretical UV–VIS spectra that were obtained from the quantum mechanical calculations (TD-DFT CAM-B3LYP/6-31 +G(d,p)) performed for the synthesized monomers agreed very well with experimentally obtained results. ^1^H NMR spectroscopy allowed us to explore the isomerization of the synthesized polymers quantitatively. Irradiation-assisted UV–VIS and ^1^H NMR spectroscopic measurements showed that the photoisomerization of azobenzene groups in the synthesized monomers and polymers is reversible.

The fabricated polymers can release energy in a solid state after the illumination of their solutions by UV light. The relatively high enthalpy of the process (17 Wh∙kg^−1^) indicates a high potential of these materials in energy storage applications. The relatively long half-life time (*τ_½_* = 3.3 days) and high activation energy (105 kJ∙mol^−1^) at room temperature indicate that the fabricated materials are suitable for long-term energy storage applications.

WAXS and POM methods revealed a liquid-crystalline behavior of the prepared polymers. The absence of crystalline peaks at wide angles and peak at ≈5° (2θ) in the WAXS diffractograms indicates a presence of nematic phase in the analyzed samples vitrified at room temperature. This peak corresponds to a characteristic correlation spacing between the mesogens in the polymer chain. The birefringent textures also evidenced the nematic order of the thin-films samples as observed under a polarized light optical microscope.

## Figures and Tables

**Table 1:** The conditions under which the polymerizations of AzoAA and AzoMA were performed.

Polymerization*	M/I/CuX/L	Catalyst	Ligand	Initiator	Solvent	Temp.	Time
						(°C)	(h)
1A	50:1:1:1	CuCl	PMDTA	ECP	Dioxane	65	24
2A		CuCl		ECP		65	48
3A		CuCl		ECP		65	72
4A		CuCl		ECP		65	96
5A		CuBr		EBriB		95	48
6A		CuBr		EBriB		95	72
1M	50:1:1:1	CuBr	PMDTA	EBriB	Dioxane	95	24
2M							48
3M							72
4M							96
5M							120
6M							132
7M	100:1:1:1	CuBr	PMDTA	EBriB	Dioxane	95	48
8M							72
9M							96
10M							120
11M							144
12M	50:1:1:1	CuBr	PMDTA	EBriB	Anisole	120	24
13M			Me_6_TREN				28
14M			Me_6_TREN				48

* Polymerizations of AzoAA are denoted as A; polymerizations of AzoMA are denoted as M.

**Table 2:** The molar ratios of reagents applied in the performed polymerizations, the time of the polymerizations, achieved monomer conversions, and characteristic of the obtained polymers from SEC analyses (number-average molecular masses; polydispersity indexes) as well as the theoretical molecular masses determined based on used molar ratios and achieved monomer conversion.

Sample	M/I/CuBr/L^a^	Temp.	Time	α^b^	M_theoretical_^c^	M_n_^d^	Đ ^e^(M_w_/M_n_)
		(°C)	(h)	(%)	(Da)	(Da)	
4A	50:1:1:1	65	96	- ^f^	- ^f^	3200	1.15
5A		95	48	- ^f^	- ^f^	1300	1.34
6A		95	72	20	2700	2600	1.37
1M	50:1:1:1	95	24	26	3700	4700	1.16
2M			48	48	6800	5900	1.24
3M			72	56	7800	6100	1.24
4M			96	78	10900	8500	1.34
5M			120	88	12300	10800	1.35
6M			132	90	12600	10700	1.31
7M	100:1:1:1	95	48	15	4200	3700	1.14
8M			72	21	5900	4700	1.17
9M			96	32	9100	10100	1.27
10M			120	47	13200	14300	1.26
11M			144	59	16400	12900	1.32
12M	50:1:1:1	120	24	32	4500	6100	1.66
13M			28	54	7500	13200	1.59
14M			48	90	12700	11900	1.60

^a^ Molar ratios of monomer (M), initiator (I), ligand (L); ^b^ *α*, conversion of monomer determined gravimetrically; α = ((M_0_ − M)/M_0_) × 100%; ^c^ M_theoretical_, theoretical molecular mass calculated as M_theoretical_ = α∙(M/I)∙M_AzoMA_; ^d^ number-average molecular weight determined from SEC; ^e^ polydispersity index determined from SEC; ^f^ conversion and theoretical molecular mass were not determined due to the negligible amount of polymer formed.

**Table 3:** Thermal and molecular characteristics of selected poly(AzoMA)s: the glass transition temperatures (*T_g_*) and nematic–isotropic phase transition temperatures (*T_NI_*) determined for the chosen poly(AzoMA)s compared with their average molecular masses (*M_n_*) and polydispersity indexes (*Đ*).

Polymer	T_g_^a^	T_NI_^b^	M_n_^c^	Đ^d^ (M_w_/M_n_)
	(°C)	(°C)	(kDa)	
7M	54	70	3.7	1.14
4M	71	87	8.5	1.34
10M	85	102	14.3	1.26

^a^ The glass transition temperatures were determined during the second cooling DSC scan at 20 K∙min^−1^; ^b^ nematic–isotropic phase transition temperatures were determined by POM technique in heating at 5 K∙min^−1^; ^c^ number-average molecular weight determined from SEC; ^d^ polydispersity indexes determined from SEC.

## Data Availability

The raw/processed data are available upon reasonable request.

## Supplementary Materials

The following are available online at https://www.mdpi.com/article/10.3390/polym13132172/s1, Figure S1: ^1^H NMR (300 MHz, CDCl_3_) spectra of selected poly(AzoMA) (sample 10M).; Figure S2: MWD curves of the synthesized polymers obtained from SEC data. The curves description corresponds to the name of the samples given in Table 1 (the central part of the manuscript); Figure S3: TGA curves recorded for the synthesized monomer AzoMA and its selected polymers obtained using CuBr/PMDETA/EBriB system with two molar ratios according to the description in Table 1 (time of polymerization is given in the legend); Figure S4: Photograph of the integrated system NMR tube and optical fiber for recording NMR spectra under illumination; Figure S5: ^1^H NMR spectra of AzoMA recorded without illumination (a), after irradiation at 365 nm (photostationary state (PSS_365_)) (b), after exposition subsequently at 460 nm (photostationary state (PSS_455_)) (c); Figure S6: DQF-gCOSY (double quantum filtered–gradient correlation spectroscopy) spectrum recorded for AzoMA; Figure S7: Eyring–Polanyi plots obtained from UV–VIS measurements of thermal isomerization *Z* → *E* AzoMA (a) and poly(AzoMA) (Sample 4M) (b). The dashed lines show the 95% confidence interval; Table S1: The thermal isomerization *Z→E* parameters determined for monomer AzoMA and selected poly(AzoMA) (Sample 4M) based on the Eyring–Polanyi equation extrapolated to 20 °C. Error margins for *ΔG^‡^* values are given as 95% confidence interval (for n-2 degrees of freedom). Error margins for *k* and *t_½_* were propagated using an exact derivative method from the fitting data; Figure S8: DSC thermogram for the solid poly(AzoMA) (Sample 10M). The data were recorded in the second heating–cooling cycle after the vitrification of the sample; Table S2: Peak positions and characteristic length scales derived from poly(AzoMA) diffractograms collected at room temperature.
