# Supplementary material for: New Photoresponsive Poly(meth)acrylates Bearing Azobenzene Moieties Obtained via ATRP Polymerization Exhibiting Liquid-Crystalline Behavior
Source: Polymers (Basel). 2021 Jun 30;13(13):2172. doi: 10.3390/polym13132172 (PMC8271449; doi:10.3390/polym13132172)
Supplement: Supplementary file 1 [file polymers-13-02172-s001.zip › polymers-1261994-supplementary.pdf]

Supplementary Information

New Photoresponsive Poly(meth)acrylates Bearing Azobenzene Moieties Obtained via ATRP Polymerization Exhibiting Liquid-Crystalline Behavior

Piotr Cieciórski, Paweł W. Majewski and Elżbieta Megiel*

Faculty of Chemistry, University of Warsaw, Pasteura 1 Str., 02-093 Warsaw, Poland

Content:

Figure S1. ^{1}H NMR (300 MHz, $CDCl_3$) spectra of selected poly(AzoMA) (sample 10M).

Figure S2. MWD curves of the synthesised polymers obtained from SEC data. The curves description corresponds to the name of the samples given in Table 1 (the central part of the manuscript).

Figure S3. TGA curves recorded for the synthesised monomer AzoMA and its selected polymers obtained using CuBr/PMDETA/EBriB system with two molar ratios according to the description in Table 1 (time of polymerisation is given in the legend).

Figure S4. Photograph of the integrated system NMR tube and optical fibre for recording NMR spectra under illumination.

Figure S5. ^{1}H NMR spectra of AzoMA recorded without illumination (a), after irradiation at 365 nm (photostationary state (PSS_{365})) (b), after exposition subsequently at 460 nm (photostationary state (PSS_{455})) (c).

Figure S6. DQF-gCOSY (Double Quantum Filtered Gradient – Correlation Spectroscopy) spectrum recorded for AzoMA.

Figure S7. Eyring-Polanyi plots obtained from UV-VIS measurements of thermal isomerisation $Z \rightarrow E$ AzoMA (a) and poly(AzoMA) (sample 4M) (b). The dashed lines show the 95% confidence interval.

Table S1. The thermal isomerisation $Z \rightarrow E$ parameters determined for monomer AzoMA and selected poly(AzoMA) (sample 4M) based on the Eyring-Polanyi equation extrapolated to 20°C. Errors margins for $\Delta G^{\ddagger}$ values are given as 95% confidence interval (for n-2 degrees of freedom). Errors margins for k and $t_{½}$ were propagated using an exact derivative method from the fitting data.

Figure S8. DSC thermogram for the solid poly(AzoMA) (sample 10M). The dataae were recorded in the second heating-cooling cycle after the vitrification of the sample.

Table S2. Peak positions and characteristic length scales derived from poly(AzoMA) diffractograms collected at room temperature.

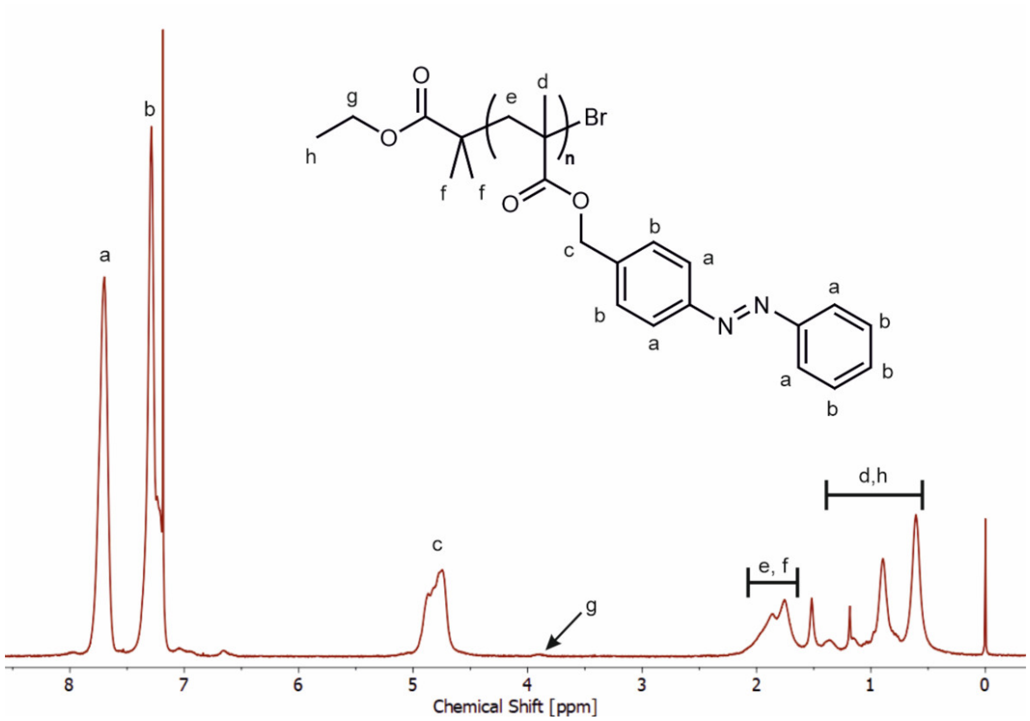

Figure S1. ^{1}H NMR (300 MHz, $CDCl_3$) spectra of selected poly(AzoMA) (sample 10M).

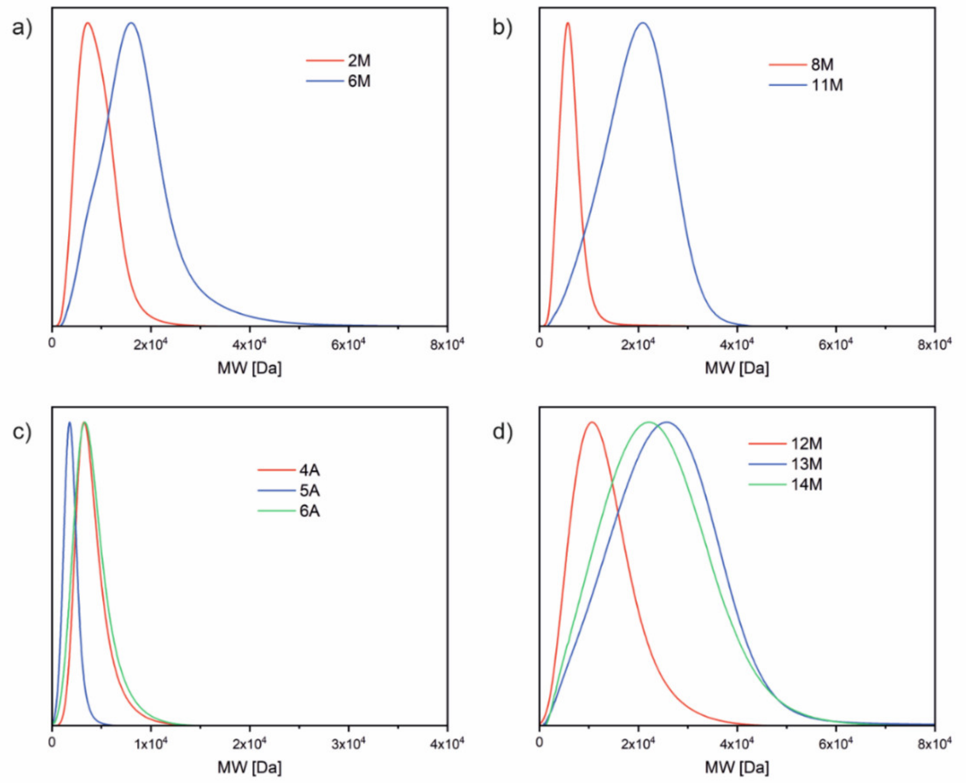

Figure S2. MWD curves of the synthesised polymers obtained from SEC data. The curves description corresponds to the name of the samples given in Table 1 (the central part of the manuscript).

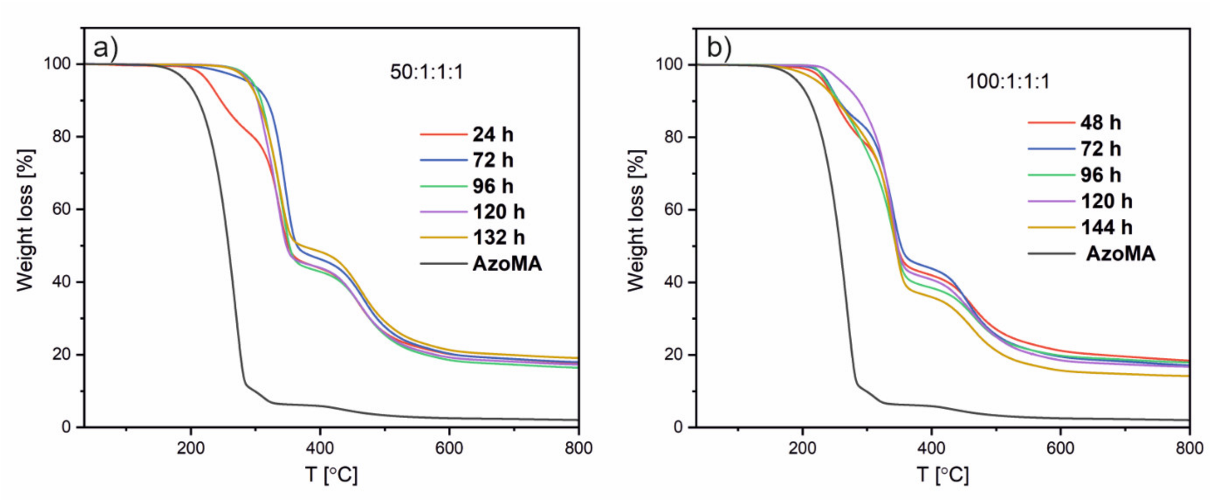

Figure S3. TGA curves recorded for the synthesised monomer AzoMA and its selected polymers obtained using CuBr/PMDETA/EBriB system with two molar ratios according to the description in Table 1 (time of polymerisation is given in the legend).

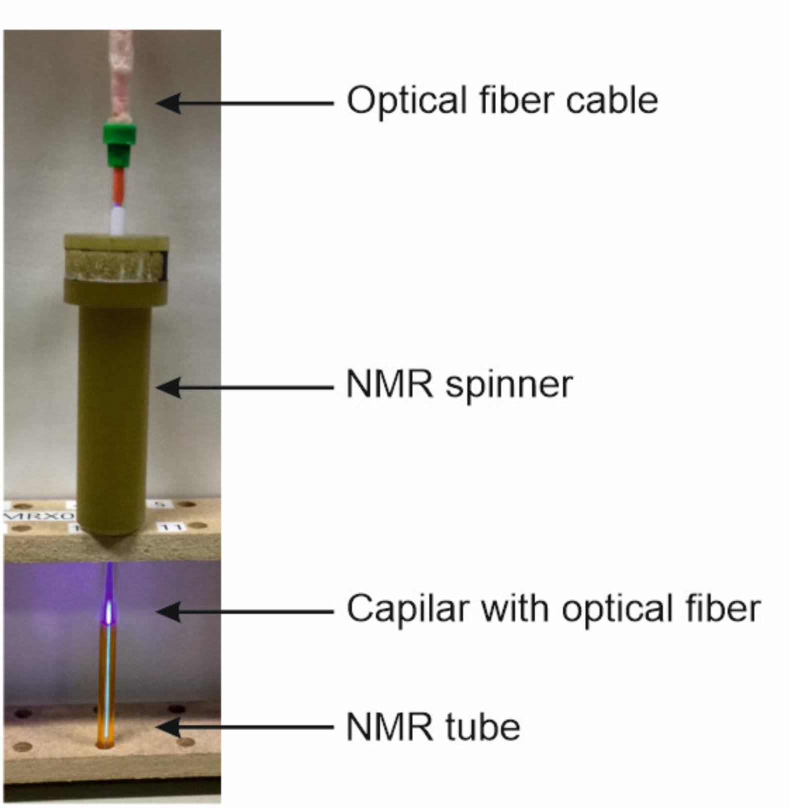

Figure S4. Photograph of the integrated system NMR tube and optical fibre for recording NMR spectra under illumination.

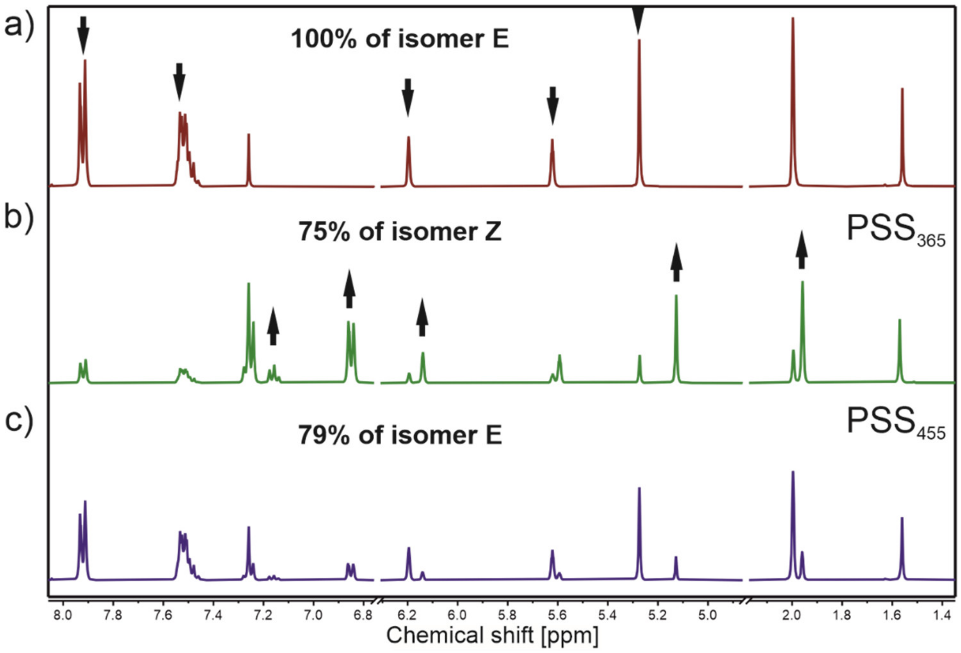

Figure S5. ^{1}H NMR spectra of AzoMA recorded without illumination (a), after irradiation at 365 nm (photostationary state (PSS_{365})) (b), after exposition subsequently at 460 nm (photostationary state (PSS_{455})) (c).

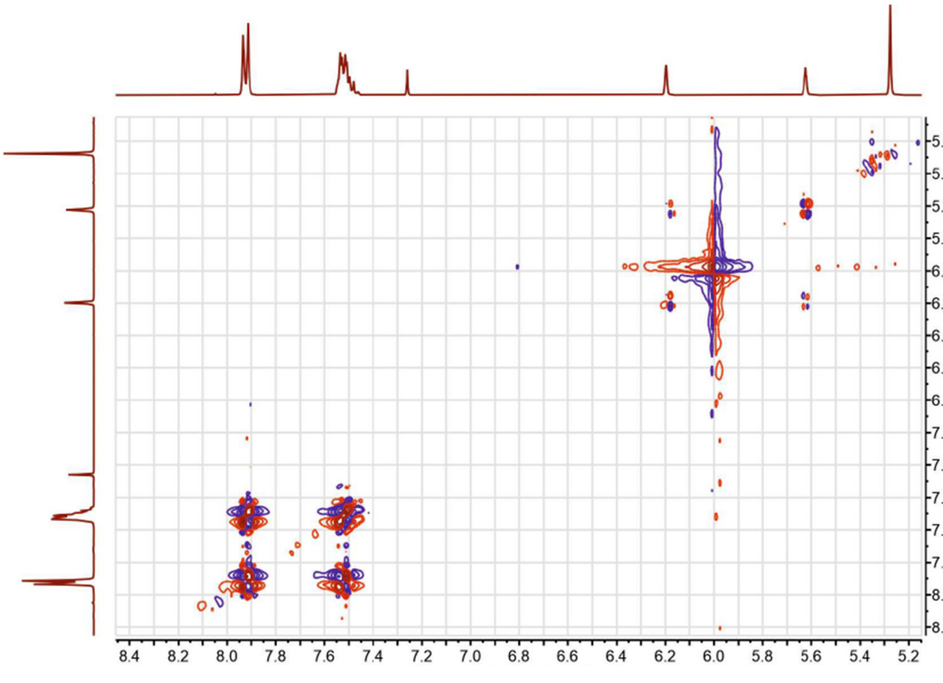

Figure S6. DQF-gCOSY (Double Quantum Filtered Gradient – Correlation Spectroscopy) spectrum recorded for AzoMA.

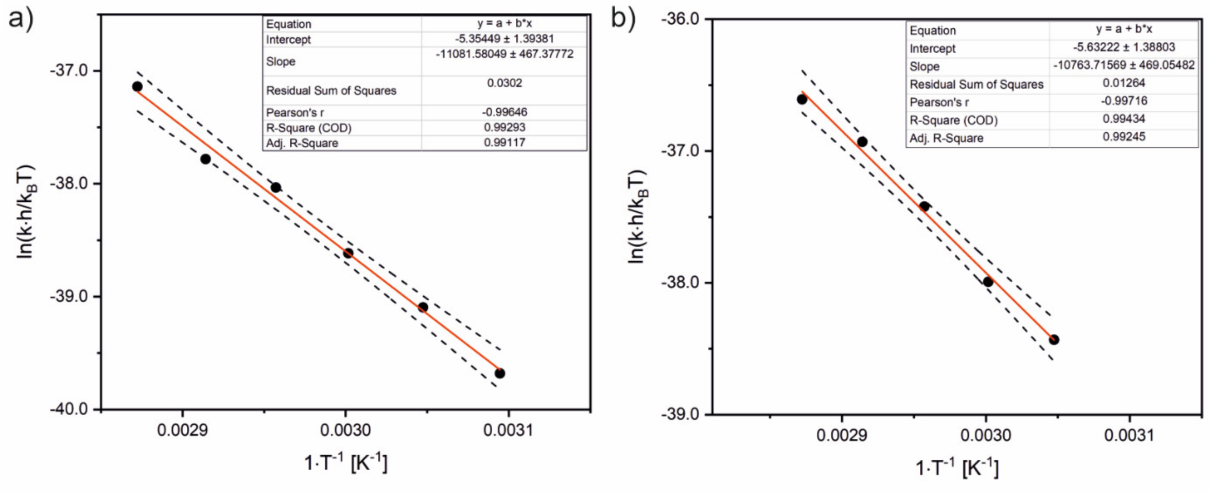

Figure S7. Eyring-Polanyi plots obtained from UV-VIS measurements of thermal isomerisation *Z* → *E* AzoMA (a) and poly(AzoMA) (sample 4M) (b). The dashed lines show the 95% confidence interval.

Table S1. The thermal isomerisation *Z* → *E* parameters determined for monomer AzoMA and selected poly(AzoMA) (sample 4M) based on the Eyring-Polanyi equation extrapolated to 20°C. Errors margins for $\Delta G^{\ddagger}$ values are given as 95% confidence interval (for n-2 degrees of freedom). Errors margins for *k* and $t_{½}$ were propagated using an exact derivative method from the fitting data.

	$\Delta G^{\ddagger}$	$k \cdot 10^{-6}$	$t_{½}$
	**[kJ·mol^{-1}]**	**[s^{-1}]**	**[days]**
**AzoMA**	105.2±1.4	1.09±0.09	7.3±0.6
**Poly(AzoMA) / 4M**	103.2±1.7	2.45±0.59	3.3±0.2

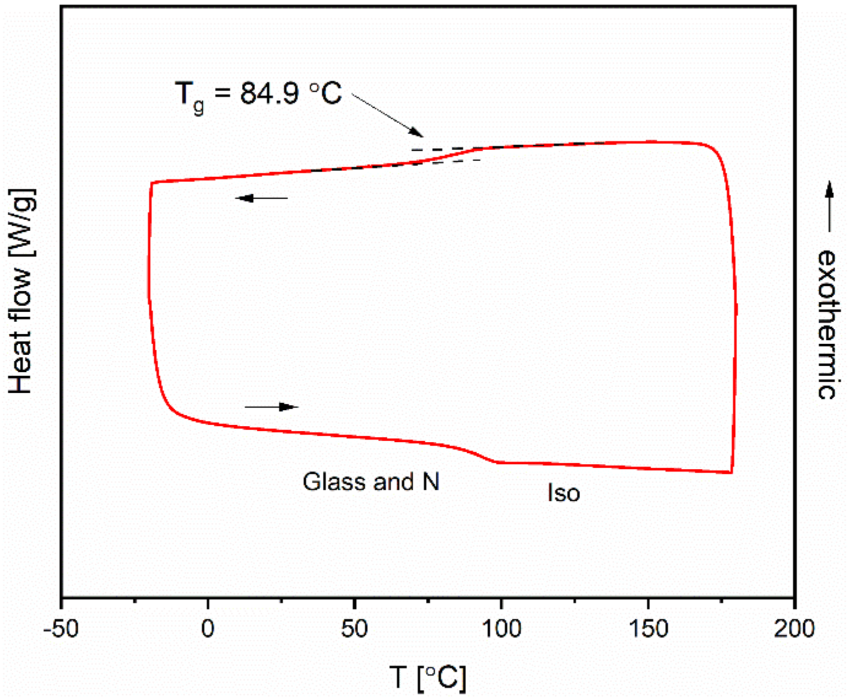

Figure S8. DSC thermogram for the solid poly(AzoMA) (sample 10M). The date was recorded in the second heating-cooling cycle after the vitrification of the sample.

Table S2. Peak positions and characteristic length scales derived from poly(AzoMA) diffractograms collected at room temperature.

	**Peak LC**	**Peak W**
	**2θ [°] (d [Å])**	**2θ [°] (d [Å])**
**Poly(AzoMA) / 7M**	4.9 (18.1)	19.9 (4.5)
**Poly(AzoMA) / 4M**	4.7 (18.9)	18.1 (4.9)
**Poly(AzoMA) / 10M**	4.6 (19.2)	18.2 (4.9)
